# A MXene‐Based Nanothermal Knife Inhibits Aggresome‐Mediated Persister Formation for Preventing Dental Caries

**DOI:** 10.1002/advs.202501501

**Published:** 2025-06-10

**Authors:** Yinyin Zhang, Leilei Yang, Jing jiao, Wenshuai Li, Sen Lin, Xianlong Zong, Haoyang Qin, Danfeng Liu, Rui Li

**Affiliations:** ^1^ Department of Stomatology The First Affiliated Hospital of Zhengzhou University Zhengzhou 45000 China

**Keywords:** early childhood caries, heat shock protein, multispecies biofilms, persister, photothermal therapy

## Abstract

Early childhood caries (ECC) is one of the most common chronic diseases among children globally, affecting the health and quality of life of children. Despite various treatments, cariogenic bacteria persist, resulting in a pessimistic outlook for the prevention and treatment of ECC. A notable presence of persister cells is found within the cariogenic bacterial population, with their dormancy enabling them to evade antiseptics. Mechanistically, the downregulation of heat shock proteins (HSPs) leads to protein aggresome formation, converting active cariogenic bacteria into persisters, which confers dual defensive barriers through metabolic inertness and concomitant reduction of membrane permeability, rendering conventional antimicrobials ineffective. Given these findings, Ti_3_C_2_ MXene, a 2D nanomaterial acting as a “nanothermal knife” to disrupt persister cell membranes is developed. Ti_3_C_2_‐mediated photothermal therapy (PTT) upregulated HSPs, inhibited aggresome formation, and prevented persister dormancy, showing efficacy against planktonic and biofilm cariogenic persisters. This PTT strategy demonstrates remarkable efficacy against planktonic cariogenic persisters and multispecies biofilms. Furthermore, the in vivo establishment of aggressive caries models reveals a substantial reduction of cariogenic pathogens following combined Ti_3_C_2_‐mediated PTT and antimicrobial treatment, effectively suppressing caries progression. This light‐assisted antibacterial strategy may aid ECC prevention.

## Graphical abstract

Early childhood caries (ECC) involves cariogenic persisters that evade antimicrobial treatment. Persisters achieve this by downregulating heat shock proteins (HSPs) and formation of protein aggregates. Ti_3_C_2_ MXene‐based photothermal therapy (PTT) acts as a “nanothermal knife,” disrupting membranes, upregulating HSPs, inhibits aggregates, prevents bacterial dormancy, effectively eliminating persisters and suppressing caries in vivo, and may aid in ECC prevention.

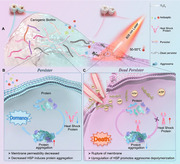

## 1 Introduction

As one of the three major diseases affecting human health, dental caries affects individuals across all age groups, with a prevalence ranging from 49% to 83%.^[^ ^1^, ^2^, ^3^ ^]^ Early childhood caries (ECC) is an aggressive and rapidly advancing form of dental caries. The ensuing pulp and periapical diseases can trigger swelling and pain in the jaw and facial regions, profoundly affecting the child's ability to chew, overall nutritional intake, craniofacial development, and even mental health.^[^ ^4^, ^5^ ^]^ The colonization of cariogenic bacteria on tooth surfaces is the main cause of caries, leading to enamel demineralization and structural damage, and promoting the development of dental caries.^[^ ^6^ ^]^ Cariogenic bacteria adhering to teeth typically exist in biofilms.^[^ ^7^ ^]^ Hence, it is essential to eradicate cariogenic bacteria and biofilms to prevent the onset of ECC.

Children often exhibit poor compliance with regular oral hygiene practices, resulting in limited effectiveness of the mechanical removal of cariogenic bacteria. Thus, broad‐spectrum antiseptics are frequently used as pharmaceutical interventions to manage cariogenic bacterial biofilms. Although these agents are effective in killing cariogenic bacteria, the cariogenic population can quickly bounce back after the removal of antiseptics.^[^ ^8^ ^]^ To investigate the underlying mechanisms, we performed in vitro experiments, which unexpectedly showed that large amounts of cariogenic bacteria survived even under extremely high concentrations of antiseptics. The bacteria that survived the action of high doses of antimicrobial agents were further demonstrated to exhibit the characteristics of persister cells, able to rapidly proliferate and continue to be pathogenic after the removal of antimicrobial agents. Persister cells are refractory bacteria that can tolerate lethal concentrations of antimicrobial agents, representing a form of phenotypic tolerance in which the progeny shares the same genetic material as the parent strain.^[^ ^9^ ^]^ By entering a dormant state, persister cells inhibit the activity of antimicrobial targets, leading to decreased bacterial susceptibility to antimicrobials and eventually evading their bactericidal effects. However, upon removing antimicrobial agents, persister cells regain their growth vigor and reproduce actively, leading to treatment failure and relapse of infectious diseases in clinical settings.^[^ ^10^, ^11^ ^]^ Moreover, the emergence of persister cells could serve as a reservoir for mutant genes, which allows single mutations to be preserved after antimicrobial treatment, enabling the accumulation of drug‐resistant mutations within bacteria over time, which leads to the development of drug‐resistant bacteria or even “superbugs,” posing significant challenges to the clinical treatment of infectious diseases.^[^ ^12^ ^]^ Therefore, it is urgently necessary to investigate the mechanism underlying persister formation, which is critical for developing new therapeutic strategies for targeting cariogenic persisters and preventing ECC.

**Scheme 1:**
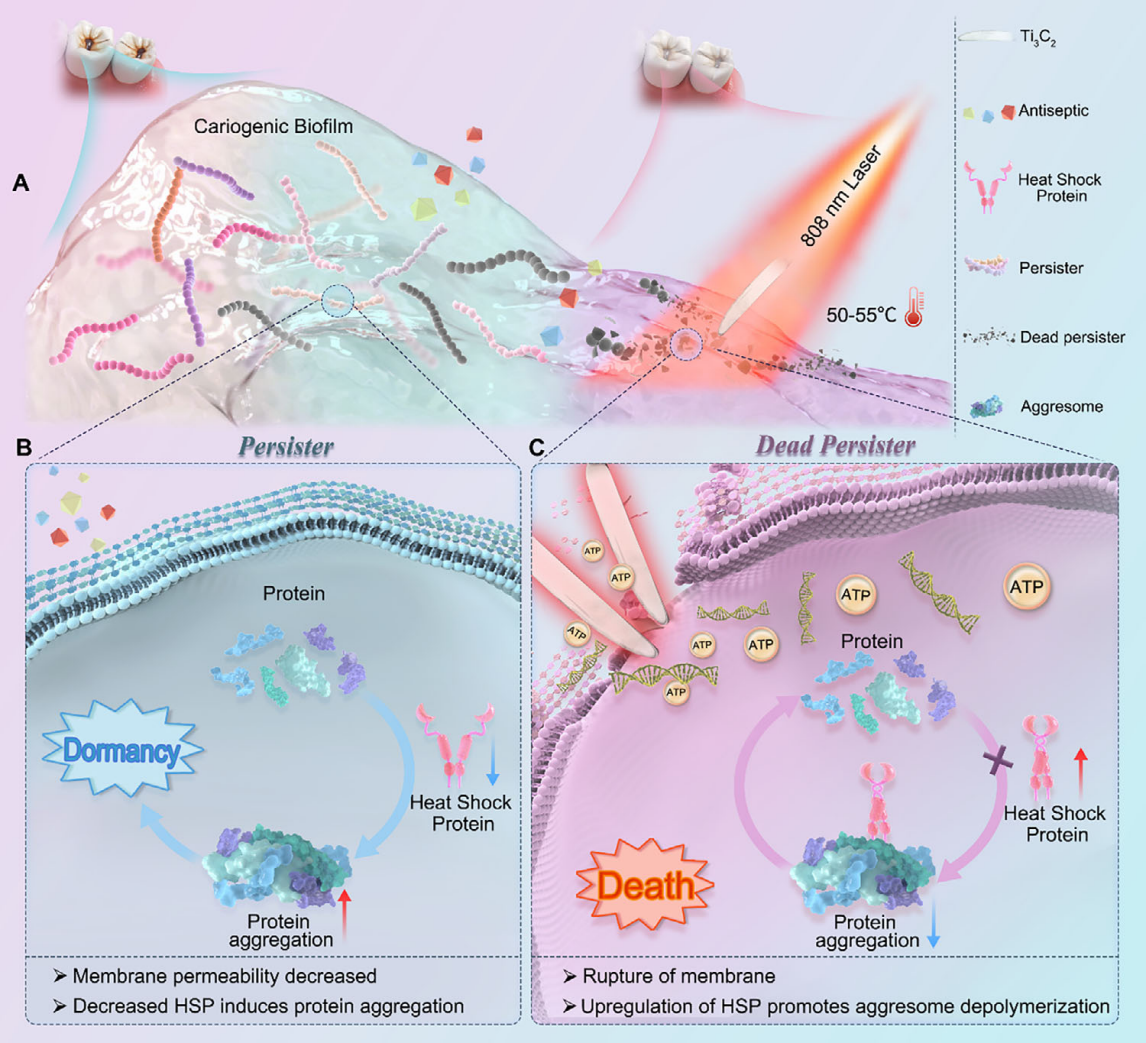
Illustration of the Ti_3_C_2_‐mediated PTT to eliminate cariogenic persister to prevent ECC. A) Cariogenic bacteria and biofilm are the leading etiology of ECC. B) Under the influence of antiseptics, proteins within bacterial cells that maintain normal life activities become misfolded and aggregate, forming PA. This inhibits bacterial metabolism, causing the bacteria to enter a dormant state and become persister cells. Decreased membrane permeability is another characteristic that protects cariogenic persister bacteria from the internalization and killing effects of antiseptics. C) PTT facilitated by this “nanothermal knife” (Ti_3_C_2_ MXene‐mediated PTT) can stimulate the upregulation of HSPs. These proteins then directly bind to and depolymerize PA, blocking the activities of persister cells. Moreover, this “nanothermal knife” can disrupt the membranes of persister cells, leading to the leakage of ATP and nucleic acids and the demise of these persister cells.

Under adverse conditions, proteins within the bacterial cells undergo misfolding and aggregation, leading to the formation of protein aggresome (PA). This process can result in a reduced metabolic rate in bacteria, facilitating the emergence of dominant persister cells.^[^ ^13^ ^]^ Heat shock proteins (HSPs) play a central role in regulating protein homeostasis and preventing protein aggregation.^[^ ^14^ ^]^ Our study revealed the significance of HSP‐PA signaling in the development of cariogenic persisters. Furthermore, we discovered that decreased membrane permeability is a crucial feature that shields cariogenic persisters from internalization and subsequent killing by antiseptics. Building on these insights, we engineered a novel 2D nanomaterial, Ti_3_C_2_ MXene, characterized by sharp knife‐like edges and exceptional photothermal therapy (PTT) properties.^[^ ^15^, ^16^ ^]^ This “nanothermal knife” can disrupt the membranes of persister cells.^[^ ^17^, ^18^ ^]^ It facilitates PTT to thwart the formation of persisters by interrupting the HSP‐PA signaling pathway. Additionally, we have assessed the efficacy of the “nanothermal knife” in eliminating persisters in planktonic cariogenic bacterial populations and within biofilms. Notably, the in vivo therapeutic efficiency and biocompatibility of Ti_3_C_2_ MXene‐mediated PTT were evaluated by establishing an aggressive ECC caries model (**Scheme** **1**). The findings of this study could provide insight into developing new approaches to improve ECC treatment.

## 2 Results and Discussion

### 2.1 Cariogenic Persister Survives the Killing of High Concentrations of Antiseptics

**Figure 1:**
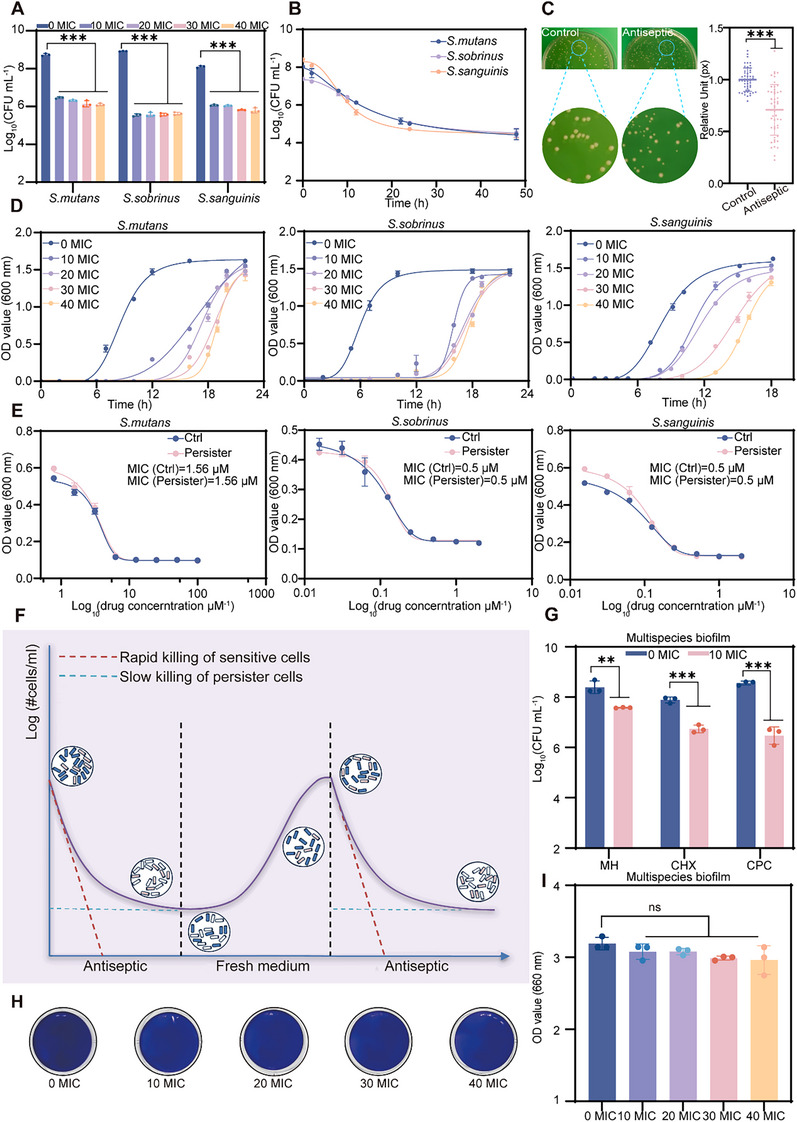
Cariogenic persister survives antiseptic treatment. A) Quantification results showing the number of surviving cariogenic bacteria after various concentrations of antiseptic treatment (MH was used). MIC indicates minimum inhibitory concentration. B) Bactericidal efficacy curves of cariogenic bacteria subjected to 40 MIC of MH. C) Comparison of bacterial colony sizes between Control and MH‐treated groups. D) The growth curve of different bacteria species after the removal of different concentrations of MH. E) Assessment and comparison of the MIC between Control and MH‐treated groups. F) The diagram illustrates the killing and regrowth kinetics of cariogenic bacterial populations under antiseptics treatment. Pink bacteria indicate persister cells, blue bacteria represent sensitive cells, and white bacteria indicate dead cells. G) Colony‐forming units (CFU) counts of bacteria in multispecies mixed biofilms following high concentrations of antiseptics. H) Violet staining indicates the biofilm formation ability of persisters 24 h the removal of antiseptic. I) Quantification of crystal violet staining results. The data are presented as mean ± standard deviation (SD) with *n* = 3; ^**^ *p* < 0.01, ^***^ *p* < 0.001, ns, *p* > 0.05.

In addition to antibiotic resistance, persistent infections present a significant challenge to the management of bacterial infections.^[^ ^13^ ^]^ Individuals with persistent infections experience either continuous or recurrent episodes of bacterial infection, often demonstrating a poor response to antibiotic therapy. Antiseptics typically target active bacterial metabolic processes; however persistent bacteria evade these treatments by entering a dormant phase marked by growth arrest. This dormancy significantly lowers metabolic activity, thereby reducing the effectiveness of antibiotics at their intended targets. This mechanism results in a decreased sensitivity to antiseptics, allowing them to evade their lethal effects.^[^ ^19^ ^]^ Nevertheless, once antiseptics are removed, persistent bacteria can regain their vitality and multiply, leading to clinical treatment failure and delayed recurrence of infectious diseases.^[^ ^20^ ^]^ First, we evaluated three clinically prevalent oral antiseptics‐minocycline hydrochloride (MH), chlorhexidine (CHX), and cetylpyridinium chloride (CPC)‐by determining the minimum inhibitory concentrations (MICs) against key cariogenic bacteria, including *Streptococcus mutans* (*S. mutans*), *Streptococcus sobrinus* (*S. sobrinus*), and *Streptococcus sanguinis* (*S. sanguinis*) (Figure S1, Supporting Information). The cariogenic bacteria were then treated with MH at concentrations of 10 times MIC, 20 MIC, 30 MIC, and 40 MIC. Compared with the untreated group, the number of cariogenic bacteria was significantly reduced after treatment with a high concentration of MH, but a large number of bacteria still survived (**Figure** **1**A; Figure S2, Supporting Information). We then tested the time‐dependent killing of cariogenic bacteria under 40 MIC of MH and observed that it exhibited a biphasic bactericidal curve (Figure 1B; Figure S3, Supporting Information), the characteristic feature of antibiotic persisters is the swift elimination of non‐persister cells, leading to a survival plateau where the tolerant persisters remain. Further comparison of colony morphology revealed that the bacteria that survived treatment with a high concentration of MH exhibited noticeable differences in size, with varying sizes, and were generally smaller (Figure 1C). After the removal of the high concentration of antiseptics and the addition of fresh medium, the bacteria were able to resume proliferation, but the growth rate was inconsistent (Figure 1D), indicating that the dormancy depth of persisters varied under different treatment groups. Persisters exhibited phenotypic tolerance with no change in MIC.^[^ ^13^ ^]^ Consistently, the microbial MIC of these viable bacteria was consistently identical to that of the susceptible bacteria (Figure 1E). High concentrations of antiseptic can rapidly kill numerous susceptible bacteria. Some bacteria can evade bactericidal effects and survive, demonstrating the characteristics of a biphasic killing curve. Upon removal of the antimicrobial agent and the addition of fresh medium, these persister cells began to revive and grow, ultimately yielding bacterial populations similar to the original flora (Figure 1F).

Planktonic bacteria encapsulated within extracellular polymeric substances constitute a highly stable and intricate ecosystem characterized by biofilms. To investigate the presence of persister cells within biofilms, we constructed a multispecies mixed biofilm model incorporating *S. mutans*, *S. sobrinus*, and *S. sanguinis*, which were then treated with various antiseptics. The quantification results revealed that at high concentrations of antiseptics, a substantial number of persisters still survived, with their proportion notably surpassing that observed in the planktonic bacterial population (Figure 1G; Figure S4, Supporting Information). Furthermore, upon withdrawal of the antiseptic, the persister cells rapidly proliferated, leading to the reformation of dense biofilm structures (Figure 1H,I). The high expression levels of cariogenic virulence genes indicated that the persister cells maintained a high potential for caries after regrowth (Figure S5, Supporting Information). These findings indicate that a single antiseptic has a limited antibacterial effect, and the presence of cariogenic persistent bacteria suggests the urgent need to better understand the chronic nature of dental caries and explore new therapeutic strategies.

### 2.2 Cunning Survival Mechanisms of Cariogenic Persisters: Decreased Membrane Permeability and Formation of Aggregates

**Figure 2:**
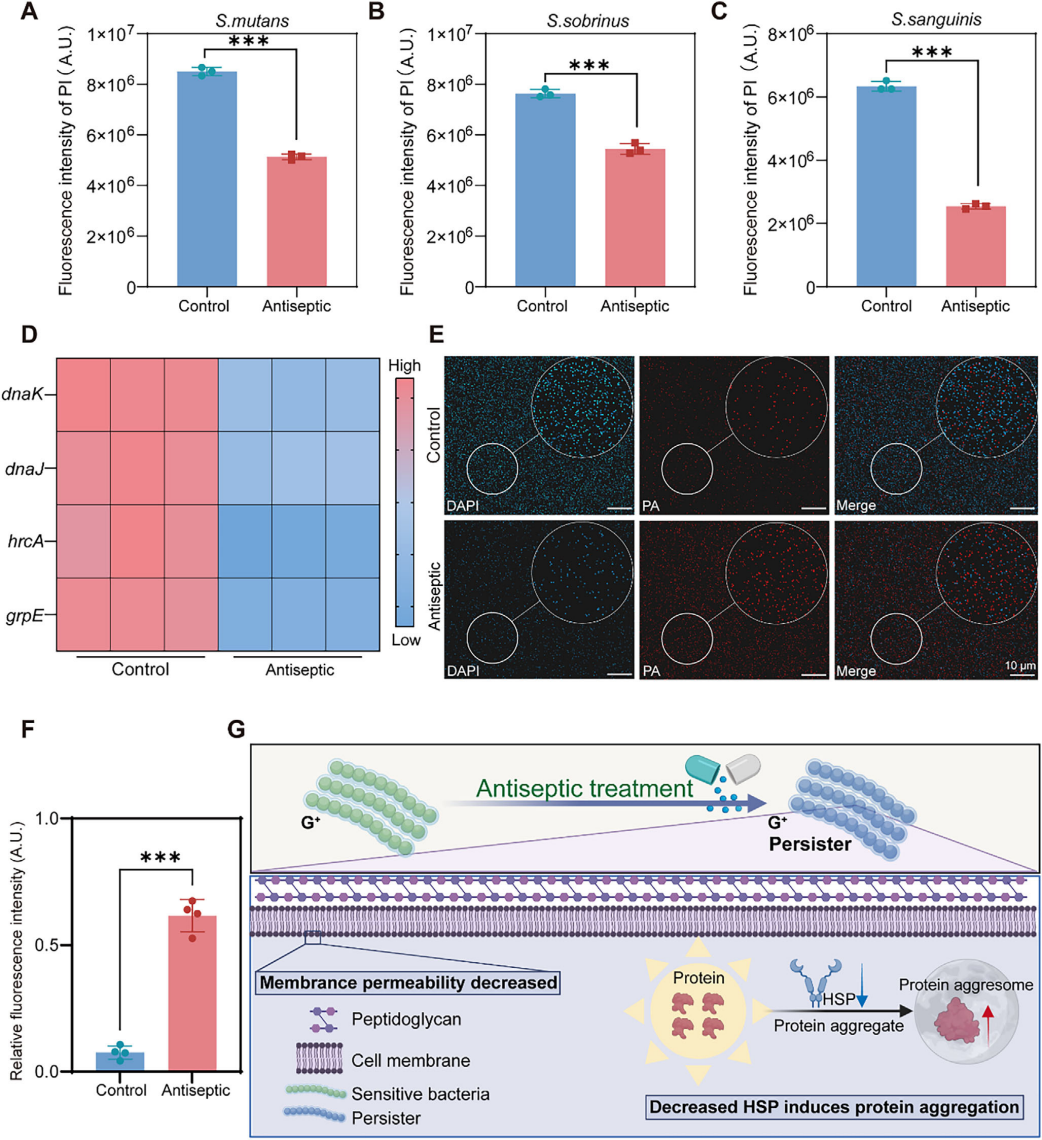
The alteration in membrane permeability and the formation of PA are intricately connected to the development of cariogenic persisters. A–C) PI fluorescence quantification of the Control and antiseptics‐treated groups. D) Heatmap showed that the expression levels of HSP‐related genes (*hrcA*, *grpE*, *dnaJ*, and *dnaK*) are significantly down‐regulated. (blue indicates low expression levels, and red indicates high expression levels). E) CLSM images detecting PA formation (blue: DAPI; red: PA). F) Quantification results showed the PA change in the Control and Antiseptic groups. (Control, treatment with Brain‐Heart Infusion Broth (BHI); Antiseptic, treatment with 40 MIC of MH). G) The schematic diagram elucidates the basic mechanisms involved in the development of cariogenic persisters. Initially, during the formation of persisters, cariogenic bacteria decrease their membrane permeability to impede the ingress of antiseptics. Subsequently, these bacteria downregulate HSPs, triggering the aggregation of proteins into PA consequently inducing a dormant state in the bacteria. This dormancy diminishes the susceptibility of antimicrobial targets, enabling persisters to evade the elimination of antiseptics. Data are presented as mean ± SD with *n* = 3; ^**^ *p* < 0.01, ^***^ *p* < 0.001.

Next, we conducted an in‐depth investigation of the mechanisms underlying the formation of cariogenic persisters. Initially, we assessed the differences in cell membrane permeability between persister and sensitive cells. Our findings indicated that the cell membrane permeability of the persister cells was significantly diminished (**Figure** **2**A–C), suggesting that the entry of the drug into persistent bacteria was inferior to that observed in sensitive bacteria. The molecular mechanisms underlying persister dormancy are diverse and include the toxin‐antitoxin (TA) modules,^[^ ^21^ ^]^ oxidative stress response pathways,^[^ ^22^ ^]^ efflux pumps and transport systems,^[^ ^23^ ^]^ epigenetic modifications,^[^ ^24^ ^]^ stringent response,^[^ ^25^ ^]^ among others. Notably, the stringent response is another well‐studied mechanism involved in bacterial cell dormancy. The dormant state of persistent bacteria leads to a significant slowdown or complete cessation of metabolic processes, enabling them to survive under harsh conditions. Generally, most proteins’ folding and aggregation states exist in a dynamic equilibrium. Changes in environmental conditions can affect both the folding and aggregation states of proteins.^[^ ^26^ ^]^ The formation of protein aggregates results in the sequestration of numerous proteins that are vital for cellular functions. This process may lead to the cessation of various biological activities, resulting in the cells entering a quiescent state.^[^ ^14^ ^]^ The formation of protein aggregates can reduce the efficacy of drugs, which is not conducive to the lethality of medication.^[^ ^27^, ^28^ ^]^ HSPs belong to a class of proteins that play crucial roles in the folding, assembly, transportation, and degradation of other polypeptides within cells.^[^ ^29^, ^30^ ^]^ Research has demonstrated that the HSP gene family, which encompasses key members, such as *hrcA*, *grpE*, *dnaK*, and *dnaJ*, constitutes an essential and omnipresent framework within bacteria. This family can catalyze the depolymerization of PA, reinstate proteins to their native conformations, and avert the transition of persister cells into a dormant state.^[^ ^14^, ^30^ ^]^ In the resting state, cell function is shut down, rendering the target of action of antibiotics ineffective and reducing the permeability of the cell membrane, which markedly diminishes susceptibility to antibiotic.^[^ ^19^ ^]^

Next, we investigated whether HSP‐PA signaling plays a vital role in the formation of cariogenic persisters. We used a mixture of three suspended bacterial strains treated with antiseptic to establish a persister model. HSP expression exhibited a declining trend in the cariogenic persisters (Figure 2D). Reduced HSP expression leads to protein aggregation,^[^ ^31^ ^]^ decreased metabolic activity, and bacterial dormancy.^[^ ^32^ ^]^ Subsequently, the fluorescence intensity of PA was assessed by confocal laser scanning microscopy (CLSM). After treatment with a high concentration of the antiseptic (Figure 2E), the Antiseptic group showed the largest red fluorescence area and highest intensity. Quantitative analysis of the red fluorescence intensity (Figure 2F) revealed a significant increase in the proportion of PA within the antibacterial agent‐treated group. These data suggest that cariogenic persisters exhibit reduced cell membrane permeability, which prevents the entry of antibacterial agents. Furthermore, under the pressure of antiseptics, cariogenic bacteria can regulate the HSP‐PA signaling pathway to enter a state of slow metabolism and proliferation rate, rendering the antiseptics ineffective (Figure 2G).

### 2.3 Ti_3_C_2_ MXene‐Mediated PTT as a Promising Strategy for Eliminating Cariogenic Persisters

**Figure 3:**
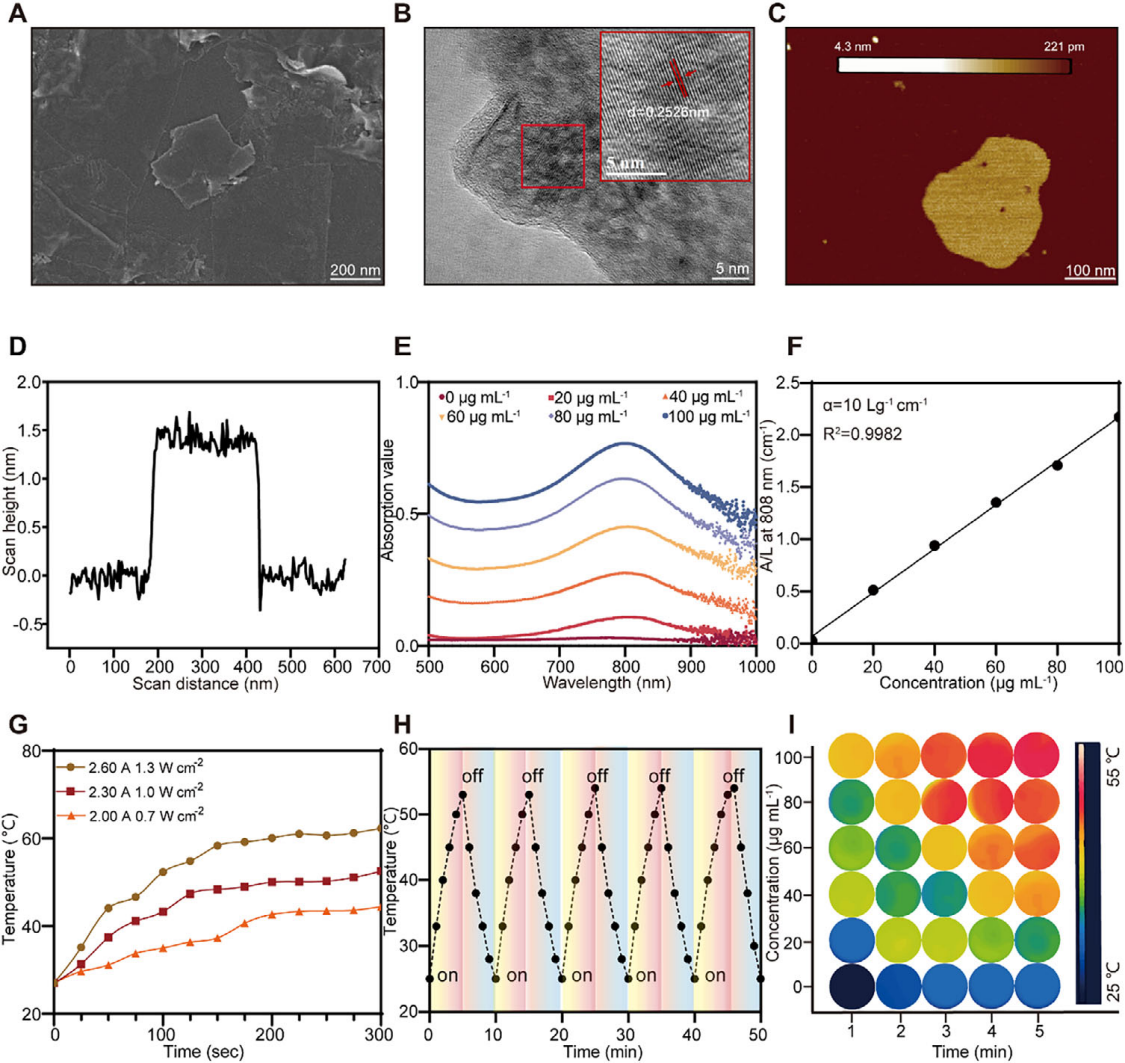
Characterization of Ti_3_C_2_. A) SEM image of Ti_3_C_2_. B) High‐resolution TEM image of crystal lattice spacing and C) AFM image of Ti_3_C_2_. D) Thickness analysis diagram of Ti_3_C_2_. E) UV–vis absorption spectra of Ti_3_C_2_. F) The absorbance intensity at 808 nm is normalized by the characteristic length of the cell (A/L) at the respective concentrations. G) Temperature variations of 100 µg mL^−1^ Ti_3_C_2_ during 5‐min laser irradiation at varying power levels. H) Photostability of Ti_3_C_2_ under 808 nm laser irradiation for five cycles (1.0 W cm^−2^). I) Photothermal images of Ti_3_C_2_ with different concentrations exposed to 808 nm laser irradiation (1.0 W cm^−2^, 5 min).

As demonstrated above, a reduction in membrane permeability is a significant factor that shuts out antiseptics, contributing to the ineffectiveness of antibacterial treatment. Thus, strategies that increase membrane permeability are the first step vital for killing persister. Furthermore, the HSP‐PA signaling cascade, which induces a dormant state in persister, attenuates their metabolic activity, rendering them less vulnerable to antibacterial assault.^[^ ^14^ ^]^ Consequently, modulating the expression of HSP could be a pivotal strategy for curbing the development of persister. This prevents active bacteria from transitioning into a dormant state, thereby enhancing the susceptibility of antibacterial targets to therapeutic interventions. Based on the dormancy mechanism of cariogenic persisters and the changes in membrane permeability, we focused our attention on 2D to prevent the side effects caused by the misuse of antibiotics. Various emerging 2D materials have shown potential in combating bacteria, such as transition metal dichalcogenides, graphene, black phosphorus, and MXenes.^[^ ^33^, ^34^ ^]^ After an extensive literature review, Ti_3_C_2_ MXene nanosheets exhibit potential antibacterial activity due to their excellent photothermal conversion efficiency and physical membrane disruption ability.^[^ ^35^ ^]^ This study innovatively proposes a synergistic strategy of combining Ti_3_C_2_ MXene‐mediate PTT with an antibacterial agent, which can overcome the inherent limitations of single therapy, such as preventing the emergence of drug‐resistant strains caused by excessive use of an antibacterial agent, the difficulty of the physical bactericidal method of MXene photothermal antibacterial in breaking through the barrier of biofilms, and the requirements of high local power and high concentration.^[^ ^36^ ^]^ We hypothesize that this therapeutic approach could effectively inhibit the formation of cariogenic persistent bacteria by increasing membrane permeability through membrane disruption and regulating the HSP‐PA signaling pathway, thereby enhancing the bactericidal effect of the antibacterial agent. Ti_3_C_2_ MXenes, which are novel 2D nanomaterials first synthesized by Gogotsi et al., have attracted extensive research attention.^[^ ^37^ ^]^ This study reports the synthesis of ultrathin 2D Ti_3_C_2_ MXene using a three‐step exfoliation process. Characterization using high‐resolution transmission electron microscopy (TEM), field‐emission scanning electron microscopy (FE‐SEM), and atomic force microscopy (AFM) confirmed the 2D lamellar structure of Ti_3_C_2_ MXene (**Figure** **3**A–C), ≈1.5 nm and particle sizes within the 500 nm range (Figure 3D). To comprehensively characterize the monolayer Ti_3_C_2_, we performed X‐ray diffraction (XRD) analysis. The characteristic peak at 39 ° corresponding to the Al atomic layer in the MAX phase (Ti_3_AlC_2_) was significantly attenuated in Ti_3_C_2_ MXene (Figure S6, Supporting Information). Furthermore, after acid etching and delamination, the (002) diffraction peak of Ti_3_C_2_ MXene exhibited a pronounced leftward shift (to lower angles), confirming the 2D nanosheet morphology of Ti_3_C_2_ MXene. We propose that the sharp, 2D “knife‐like” edges of this nanomaterial may perturb the membrane integrity of persister cells. Figure 3E depicts the characteristic absorption peaks of Ti_3_C_2_ at varying concentrations at 808 nm within the first optical window, indicating its potential as an effective photothermal agent for PTT. In accordance with the Lambert‐Beer law,^[^ ^38^ ^]^ the extinction coefficient was determined to be 10 Lg^−1 ^cm^−1^ (Figure 3F). This value exceeds that of various photothermal materials, the magnitude of the extinction coefficient can reflect the absorption efficiency of molecules for specific wavelengths of light; the larger the value, the stronger the substance's absorption capacity at that wavelength.^[^ ^39^, ^40^ ^]^ An 808 nm near‐infrared laser was applied at varying power levels to irradiate the Ti_3_C_2_ for 5 min, and an infrared thermal imager was used to monitor temperature changes and plot the heating curve (Figure 3G). The Ti_3_C_2_ reached a maximum temperature of 55 °C at a power density of 1.0 W cm^−2^. A constant power density of 1.0 W cm^−2^ was chosen for the experiments to avert thermal damage to the dental pulp and adjacent soft tissues. Moreover, the temperature cycling demonstrated a peak of ≈55 °C over five cycles, indicating that the 100 µg mL^−1^ Ti_3_C_2_ exhibited remarkable photothermal stability (Figure 3H). This ensured a sustained antibacterial effect under 808 nm laser irradiation. Photothermal images of solutions with different concentrations of Ti_3_C_2_, irradiated at a power density of 1.0 W cm^−2^ for 5 min, are presented in Figure 3I. We posit that Ti_3_C_2_ MXene acts as a “nanothermal knife,” with dual effects: its ability to disrupt the membrane structure of cariogenic persisters, and its potential to upregulate HSP and inhibit the formation of PA, preventing the development of persisters.

### 2.4 Ti_3_C_2_‐Mediated PTT for Eliminating Persisters in Planktonic Cariogenic Communities

**Figure 4:**
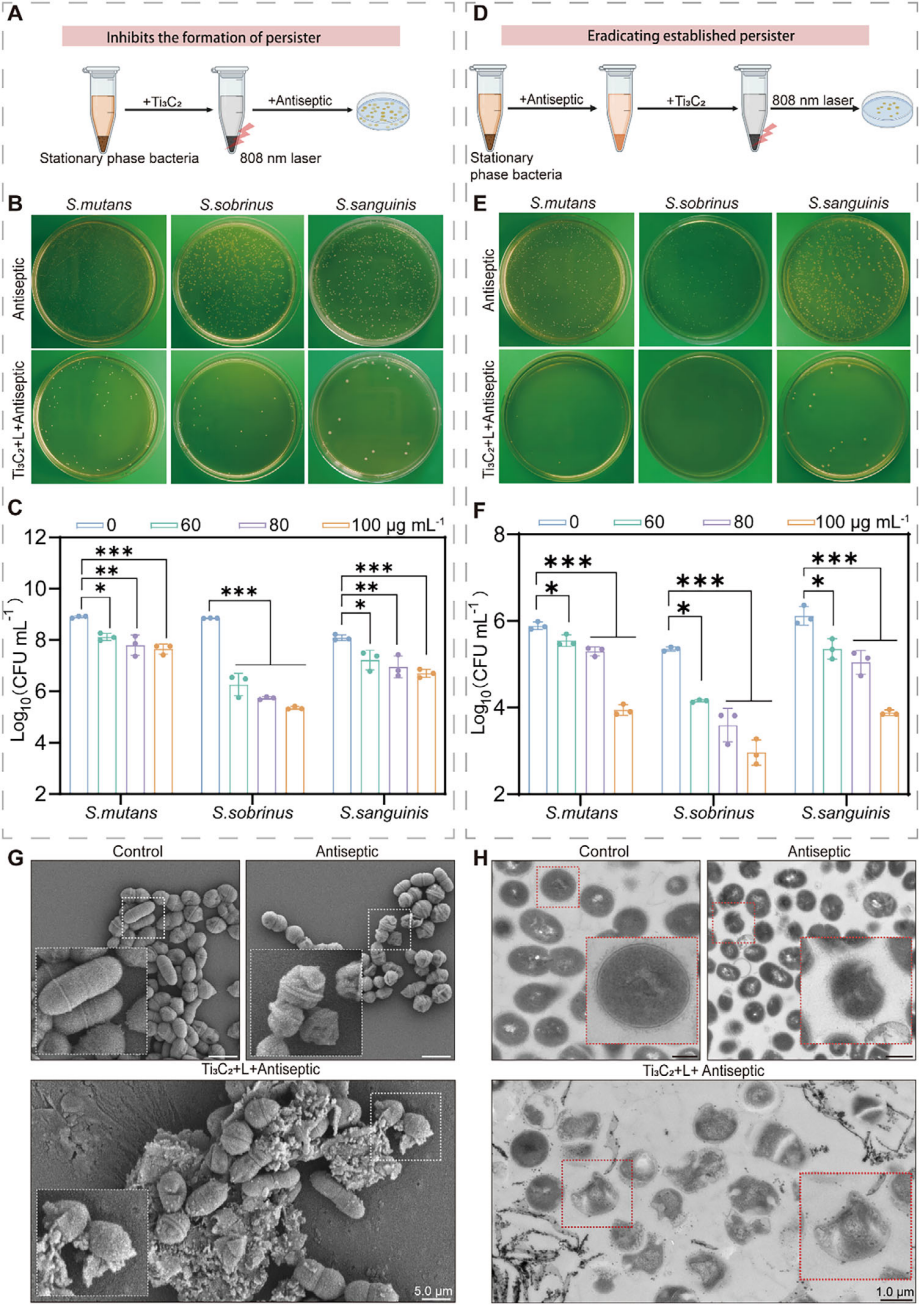
Anti‐persister efficiency of Ti_3_C_2_‐mediated PTT in vitro. A) Schematic illustration indicates that Ti_3_C_2_‐mediated PTT inhibits the formation of persistent bacteria. B) Representative photographs of bacterial colonies in the Control and Antiseptic groups (Control: BHI; Antiseptic: 10 MIC of MH). C) Inhibitory effects of different treatments on the formation of persistent bacteria. D) Schematic of Ti_3_C_2_‐mediated PTT killing of preformed persisters. E) Representative images of bacterial colonies in the Control and Antiseptic groups (Control: BHI; Antiseptic: 40 MIC of MH). F) Evaluation of the anti‐persister effect of Ti_3_C_2_‐mediated PTT on persisters. G) Morphological changes of bacteria in different treatment groups observed through SEM (the enlarged image is on the left; scale bar, 5.0 µm). H) Morphological changes of bacteria in different treatment groups observed by TEM (the enlarged image is on the right; scale bar, 1.0 µm. Control: BHI; Antiseptic: 40 MIC of MH; Ti_3_C_2_+L+Antiseptic, treatment with 40 MIC of MH and 100 µg mL^−1^ Ti_3_C_2_ upon laser irradiation). The data are presented as mean ± SD with *n* = 3; ^*^ *p* < 0.05, ^**^ *p* < 0.01, ^***^ *p* < 0.001.

We investigated the antibacterial effects of Ti_3_C_2_‐mediated PTT on planktonic cariogenic persister bacteria, focusing on two aspects: inhibition of persister cell formation and eradication of preformed persister cells. In the study of inhibiting persister formation, three cariogenic bacteria were each cultured to the stationary phase and then pretreated with Ti_3_C_2_‐mediated PTT, followed by the addition of the antiseptic MH. The results indicated that pretreatment with Ti_3_C_2_‐mediated PTT significantly enhanced the antibacterial effect of MH, with a marked reduction in the formation of persisters, and this effect was dose‐dependent (**Figure** **4**A–C; Figure S7, Supporting Information). Subsequently, to evaluate the effectiveness of eliminating established persister cells, high concentrations of MH were first used to treat the cariogenic bacteria in the stationary phase for 24 h, followed by the application of Ti_3_C_2_‐mediated PTT (Figure 4D). The results revealed that the bactericidal rate of Ti_3_C_2_‐mediated PTT against the three types of persisters could reach 99% (Figure 4E,F; Figure S8, Supporting Information).

Dental caries is essentially a result of the synergistic action of multiple bacterial species.^[^ ^6^ ^]^ A mixed bacterial suspension of three cariogenic species—*S. mutans*, *S. sobrinus*, and *S. sanguinis*— were prepared to simulate the symbiotic state of cariogenic bacteria in the oral environment. This approach allowed for a more accurate assessment of the effects of Ti_3_C_2_‐mediated PTT on disrupting complex bacterial communities. By combining SEM and TEM, we were able to directly observe changes in the ultrastructures of bacterial cells under different treatment conditions (Figure 4G,H). The observations revealed that the bacteria in the control group exhibited a regular, rounded structure with a smooth and clear surface, maintaining a complete and continuous bacterial architecture without any signs of abnormal morphology. The surface membrane of bacteria treated with 40 MIC of MH exhibited wrinkling on the cell surface membrane, a decrease in intracellular material density, weakening and rupture of the cell wall mesh structure, and the formation of cell wall‐less bacterial forms. After Ti_3_C_2_‐mediated PTT, the structural integrity of the bacteria was compromised, with cell wall rupture and separation from the cell membrane. Numerous vacuolar density images appeared within the cells, and material fragments were observed within the bacterial structures.

When observing the three bacteria separately via TEM (Figure S9, Supporting Information), we found that all three bacteria had distinguishable and intact structures in the control group. After the treatment with the antiseptic, the bacterial cell walls lost their smooth and continuous morphology, becoming irregular and wrinkled. However, after the treatment with Ti_3_C_2_‐mediated PTT, the bacterial cell membranes ruptured, and the bacteria became severely deformed, presenting an amorphous state. These phenomena indicate that Ti_3_C_2_ accelerated the physical damage to the bacterial membrane under 808 nm irradiation, destroying bacterial structures and leading to bacterial death.

### 2.5 Ti_3_C_2_‐Mediated PTT for Eliminating Persisters in Cariogenic Biofilms

During their growth and proliferation, bacteria predominantly adopt two distinct phenotypic states.^[^ ^41^ ^]^ The first is the planktonic mode, wherein individual bacterial cells exist as free‐floating, solitary entities commonly observed in liquid culture systems. These individual bacteria grow and reproduce autonomously, each acquiring nutrients and conducting metabolic activities within a suitable environment.^[^ ^42^ ^]^ The second mode involves the formation of complex, multicellular aggregates known as biofilms, wherein bacterial populations adhere to biotic or abiotic surfaces and become encased within a self‐secreted extracellular polymeric matrix.^[^ ^43^ ^]^ The dense matrix characteristics of biofilms, in conjunction with the scarcity of nutrients and oxygen, foster the emergence of persister cells. Compared to their planktonic counterparts, biofilms harbor a substantially greater number of persister cells, whose drug resistance can surpass that of planktonic organisms by a factor of 10 to 1000.^[^ ^43^, ^44^ ^]^ This pronounced increase in drug resistance complicates the task of antibiotics in eliminating biofilms, thereby amplifying the intricacy of treatment regimens.^[^ ^7^ ^]^ Consequently, there is an urgent need to assess the potential of Ti_3_C_2_‐mediated PTT in eradicating persisters within cariogenic biofilms.

**Figure 5:**
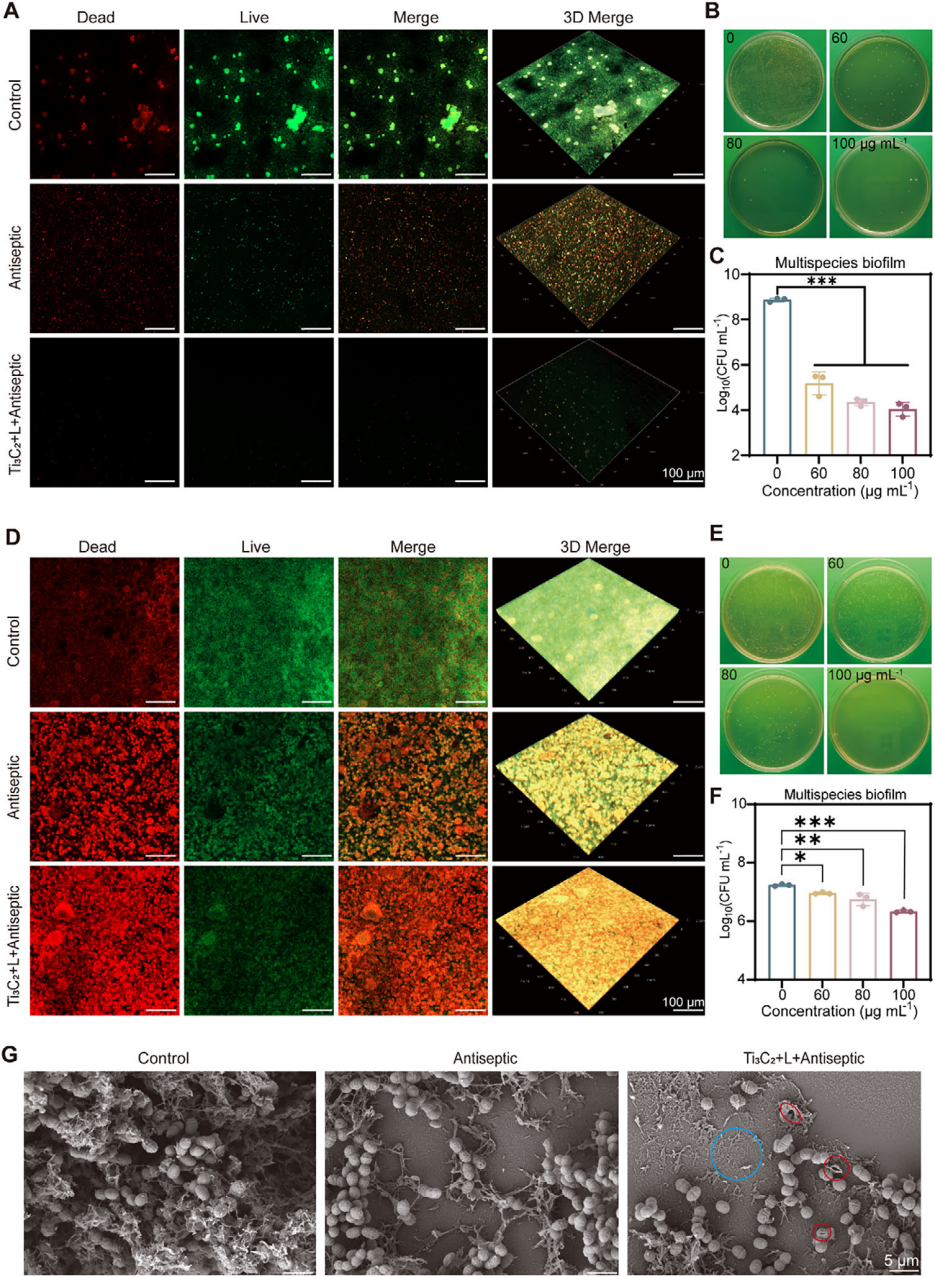
Biofilm inhibition and killing ability of Ti_3_C_2_‐mediated PTT. A) Staining of live and dead bacteria in multispecies biofilms following various treatments for the inhibition assay (scale bar, 100 µm; green, live bacteria; red, dead bacteria). B) Representative images of bacterial colonies in the Control and Ti_3_C_2_+L+Antiseptic groups (Control: BHI, Antiseptic: 10 MIC of MH, Ti_3_C_2_+L+Antiseptic: 10 MIC of MH and 100 µg mL^−1^ Ti_3_C_2_ upon laser irradiation). C) CFU counts for mixed‐species biofilm inhibition experiments. D) Staining of live and dead bacteria in multispecies biofilms following various interventions for the eradication assessment (scale bar, 100 µm; green, live bacteria; red, dead bacteria). E) Representative images of bacterial colonies in the Antiseptic and Ti_3_C_2_+L+Antiseptic groups. F) CFU counts for mixed multi‐species biofilm eradication assays (Control: BHI, Antiseptic: 40 MIC of MH, Ti_3_C_2_+L+ Antiseptic: 40 MIC of MH and 100 µg mL^−1^ Ti_3_C_2_ upon laser irradiation). G) FE‐SEM image of Ti_3_C_2_‐mediated PTT eradication of mature biofilms. (Blue circles indicate stripped biofilms, red circles denote ruptured bacteria; scale bar, 5 µm). Data are presented as mean ± SD with *n* = 3; ^*^ *p* < 0.05, ^**^ *p* < 0.01, ^***^ *p* < 0.001.

Our initial investigation focused on whether Ti_3_C_2_‐mediated PTT can effectively prevent cariogenic biofilm formations. The bacteria in the plateau phase were first exposed to Ti_3_C_2_‐mediated PTT, followed by the addition of a high concentration of MH. Live/dead staining revealed a significant reduction in biofilm quantity (**Figure** **5**A). Notably, the presence of live persisters within the biofilms was substantially decreased by Ti_3_C_2_‐mediated PTT, which was further confirmed by plate coating and quantitative analysis (Figure 5B,C). To evaluate the efficacy of Ti_3_C_2_‐mediated PTT in eliminating persisters within established biofilms, we first treated biofilms with a high concentration of MH to eliminate susceptible bacteria. The biofilms were then subjected to Ti_3_C_2_‐mediated PTT. The live/dead staining experiment indicated a remarkable decrease in live persisters (Figure 5D), which was further substantiated by plate coating and quantitative analysis (Figure 5E,F). Crystal violet staining was used to measure biofilm biomass following various treatments (Figure S10, Supporting Information). The results demonstrated a reduction in the biofilm mass within the treated groups, indicating that Ti_3_C_2_‐mediated PTT could effectively eliminate mature preformed cariogenic biofilms. Similarly, Ti_3_C_2_‐mediated PTT demonstrated significant bactericidal efficacy against persister cells within mature biofilms that had been treated with CHX and CPC (Figures S11 and S12, Supporting Information). In the biofilm samples treated with the combination of Ti_3_C_2_‐mediated PTT and antiseptic, the red fluorescent areas were more extensive, and the fluorescence intensity was enhanced.

SEM imaging (Figure 5G) revealed that cariogenic bacteria were enriched within a complex extracellular polysaccharide matrix in the control group. After treatment with a high concentration of antiseptic, there was a marked decrease in the bacterial population density within the biofilm, leading to a less compact structural arrangement. However, no significant alterations in the bacterial surface morphology were observed. In contrast, the Ti_3_C_2_‐mediated PTT group exhibited signs of bacterial surface cutting and fragmentation. These results indicate that Ti_3_C_2_‐mediated PTT can effectively inhibit the formation of multispecies biofilms and eradicate persister cells within mature biofilms.

### 2.6 Two Birds with One Stone: Ti_3_C_2_‐Mediated PTT for Disrupting Cell Membrane and Inhibiting PA Formation

**Figure 6:**
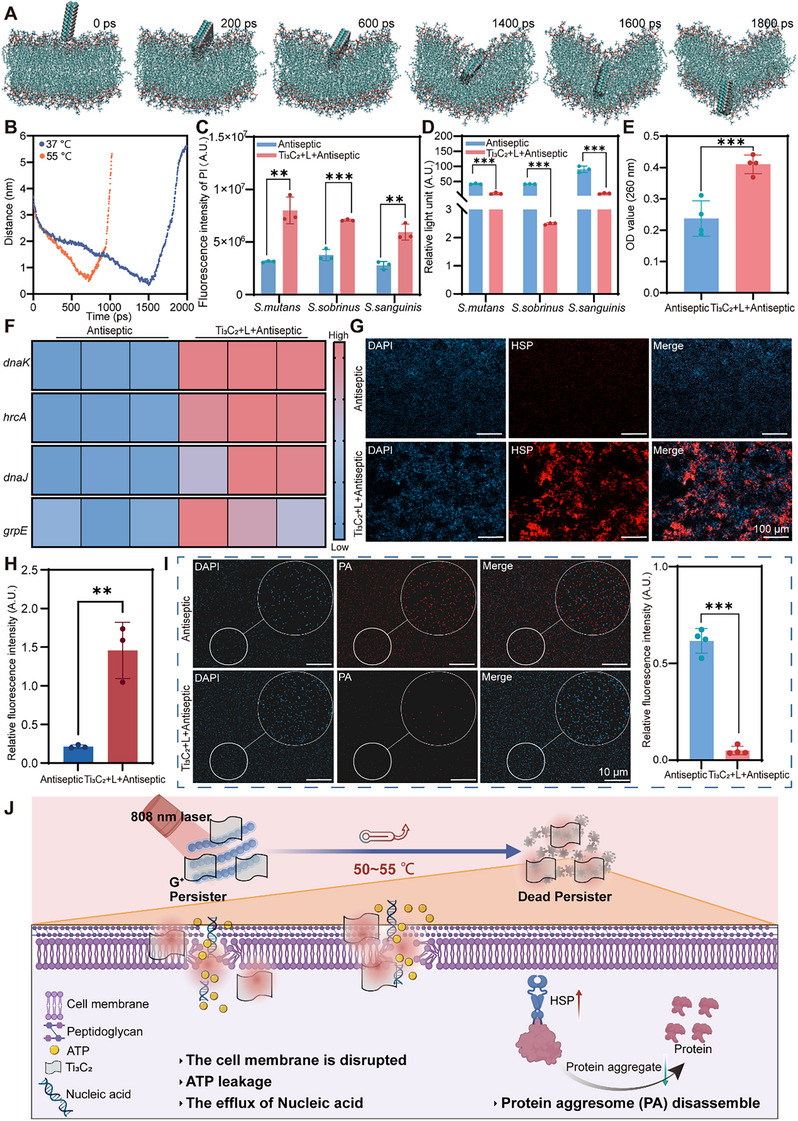
Ti_3_C_2_‐mediated PTT for disrupting cell membranes and inhibiting PA formation. A,B) Time‐dependent evolution of mass center distance between Ti_3_C_2_ surface and the lipid membrane (inset representative snapshots depicting the penetration processes). C) Quantitative PI fluorescence of persister after different treatments. D) Intracellular ATP levels following PTT treatment of cariogenic persistent bacteria. E) The effect of PTT treatments on the OD_260 nm_ of cariogenic persister indicates nucleic acid leakage. F) The expression levels of HSP‐related genes (*hrcA*, *grpE*, *dnaJ*, and *dnaK*) are significantly upregulated (Blue indicates low expression, red indicates high expression). G,H) Immunofluorescence detection of HSP expression and relative fluorescence intensity (Blue: DAPI; Red: HSP; scale bar, 100 µm). I) Representative CLSM images and quantification results detecting PA in bacteria (Blue: DAPI; Red: PA; Antiseptic, treatment with 40 MIC of MH; Ti_3_C_2_+L+Antiseptic, treatment with 40 MIC of MH and 100 µg mL^−1^ Ti_3_C_2_ upon laser irradiation). J) Illustration showing the mechanism of Ti_3_C_2_‐mediated PTT for killing persisters. The data are shown as mean ± SD with *n* = 3; ^**^ *p* < 0.01, ^***^ *p* < 0.001.

To elucidate the evolution of the interface between the Ti_3_C_2_ MXene flakes and bacterial membranes, we employed molecular dynamics (MD) simulations to investigate this process. At 37 °C, the material structure demonstrated good membrane penetration capabilities, disrupting the integrity of the lipid membrane. The invasion process was observed by measuring the centroid distance between the material surface and the lipid membrane (**Figure** **6**A). The structure contacted the exterior of the membrane at approximately 200 picoseconds (ps), subsequently penetrating the cell membrane continuously, and achieving vertical separation after 1800 ps. At 2000 ps, the vertical separation displayed a distance of ≈5.5 nm, highlighting the special ability of the knife‐like structure to disrupt the lipid membrane effectively. The material structure exhibited stronger membrane penetration capabilities at 55 °C to explore the effect of increased temperature on the material's membrane disruption rate while keeping other parameters of the simulation system constant (Figure S13, Supporting Information). The time required for the material to disrupt the membrane was significantly reduced, with the membrane disruption time being halved compared to that at 37 °C (Figure 6B). Additionally, we tracked the temporal variation in the interaction energy between the surface and the lipid membrane (Figure S14, Supporting Information). From the initiation of the penetration process to complete invasion, the binding energy between the material and the membrane gradually increased; the binding energy between the material and the membrane gradually decreased from complete invasion to detachment. Maximum binding energy was observed at the point of complete invasion, indicating the most stable state of the system. This suggested that the material tended to fully disrupt the membrane. Therefore, MD simulations confirmed that Ti_3_C_2_ disrupts bacterial membranes, with the rate of membrane disruption accelerating as temperature increases. The Ti_3_C_2_‐mediated PTT not only disrupts membrane structural integrity but also, increases the space between lipid molecules through local hyperthermia, significantly elevating membrane permeability.^[^ ^17^ ^]^ We assessed cell membrane permeability and found that the photothermal group exhibited significantly greater permeability than the Antiseptic group (Figure 6C). Additionally, we evaluated the changes in ATP levels, which are crucial for sustaining bacterial life activities, and observed substantial ATP leakage in the Ti_3_C_2_‐mediated PTT group (Figure 6D). Typically, macromolecules such as DNA and RNA, cannot traverse intact bacterial cell membranes. However, when the integrity of the bacterial membrane is compromised, these macromolecules escape into the external environment surrounding the bacterial cell. The results indicated that when the persisters were subjected to Ti_3_C_2_‐mediated PTT, the absorbance of the bacterial culture medium at 260 nm increased (Figure 6E). This observation suggests increased cell membrane damage and enhanced permeability, resulting in the continuous leakage of bacterial DNA and RNA.^[^ ^45^ ^]^

Ti_3_C_2_ exhibits excellent photothermal conversion capability, enabling rapid temperature elevation under 808 nm laser irradiation. This localized temperature increase can induce a stress response in HSPs and upregulate their expression.^[^ ^46^, ^47^ ^]^ Heatmap results showed that compared to the Antiseptic group, the Ti_3_C_2_+L+Antiseptic group exhibited a reddish hue (Figure 6F), indicating that after Ti_3_C_2_ photothermal treatment, HSP expression in persister cells was elevated. Additionally, we evaluated HSP expression in biofilms across different treatment groups. Immunofluorescence imaging showed that the antiseptic group exhibited weak and sparsely distributed red fluorescence, whereas the photothermal material group displayed significantly stronger and densely packed red fluorescence forming clump‐like clusters, indicating that Ti_3_C_2_‐mediated PTT upregulated HSP expression (Figure 6G,H). Validation via fluorescence in situ hybridization (FISH) corroborated these findings, with the Ti_3_C_2_ photothermal group demonstrating enhanced red fluorescence intensity (Figure S15, Supporting Information). These results indicate that Ti_3_C_2_‐mediated PTT can upregulate HSP expression in persister bacteria. HSP eliminates misfolded proteins and interacts with PA to facilitate their depolymerization.^[^ ^48^, ^49^ ^]^ The fluorescence intensity of PA was assessed by CLSM. Notably, the Ti_3_C_2_+L+Antiseptic group exhibited the lowest red fluorescence intensity (Figure 6I). According to the quantitative analysis of red fluorescence intensity, the proportion of protein aggregates in the Ti_3_C_2_+L+Antiseptic group was significantly reduced. Disaggregation of protein aggregates disrupts the dormancy of persistent bacteria.^[^ ^14^ ^]^ Consequently, we speculated that the anti‐persister mechanism mediated by Ti_3_C_2_‐mediated PTT might be that under photothermal action, HSP upregulation disturbs the formation and maintenance of PA, blocking the dormant state of the bacterial persisters (Figure 6J). Concurrently, the opening of the cell membrane leads to the leakage of bacterial ATP and the outflow of nucleic acids, destroying the integrity of bacterial morphology and structure and resulting in bacterial death.

### 2.7 In Vivo Evaluation of Ti_3_C_2_‐Mediated PTT for ECC

**Figure 7:**
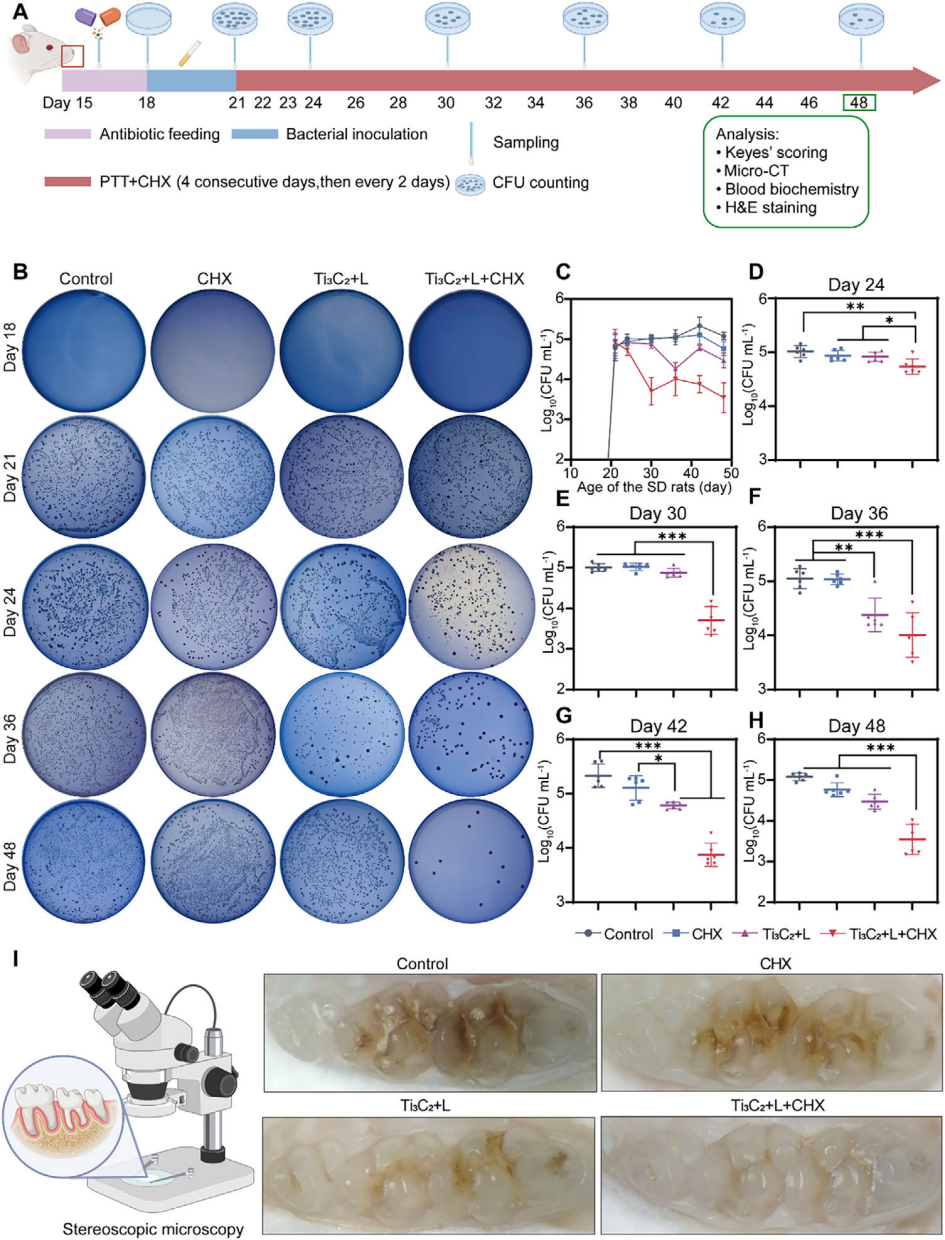
In vivo, assessment of Ti_3_C_2_‐mediated PTT combined with an antiseptic for dental caries. A) Schematic diagram of ECC model building, therapy, and assessments of anti‐caries efficacy. B) Representative images of Mitis salivarius agar (MSB) from different groups (Control: PBS, CHX: treatment with CHX, Ti_3_C_2_+L: Ti_3_C_2_ upon laser irradiation, Ti_3_C_2_+L+CHX: Ti_3_C_2_ upon laser irradiation and CHX). C) CFU counting for surviving bacterial colonies at 18, 21, 24, 30, 36, 42, and 48 days. D–H) Statistical evaluation of surviving bacterial colonies after Ti_3_C_2_‐mediated PTT combined with CHX. I) Illustration and typical photographs upon stereoscopic microscopy of the occlusal surfaces of teeth. The data are presented as mean ± SD (n = 6); ^*^ *p* < 0.05; ^**^ *p* < 0.01; ^***^ *p* < 0.001.

To further confirm the therapeutic efficacy of Ti_3_C_2_‐mediated PTT combined with antiseptic (CHX) in eliminating persister bacteria and preventing ECC in living systems, an ECC model was established using 15‐day‐old rats,^[^ ^50^ ^]^ as illustrated in **Figure** **7**A. To mitigate the effects of indigenous oral microorganisms, antibiotics were administered to rats to clear the existing oral microbial flora. Following this, cariogenic bacteria, including *S. mutans*, *S. sobrinus*, and *S. sanguinis*, were applied to the teeth for three consecutive days starting from Day 18. Subsequently, the rats were fed a diet and water enriched with sucrose to replicate the conditions of ECC.^[^ ^51^ ^]^ Ti_3_C_2_‐mediated PTT, in combination with antiseptic therapy, was initiated on Day 21 and administered for four consecutive days. The treatment regimen was adjusted every two days. Throughout the establishment of the caries model and the administration of PTT, oral sampling and CFU counts were performed at regular intervals to track the levels of cariogenic bacteria (Figure S16, Supporting Information). Following sacrifice, the carious lesions were evaluated using micro‐CT imaging and the Keyes'‐scoring system. On Day 18, during the initial sampling of the rat oral cavity, no bacterial colonies were detected on the selective medium, as shown in Figure 7B, indicating that the indigenous oral microbiota was effectively suppressed by the antibiotic treatments. Successful colonization of the oral cavity by these bacteria was observed on Day 21 after three consecutive days of inoculation with cariogenic bacteria. Remarkably, the combination of Ti_3_C_2_‐mediated PTT and antiseptic therapy resulted in a 90% reduction in the growth of cariogenic bacteria (Figure S17, Supporting Information). The results of targeted sampling and the statistical significance of antibacterial efficacy across the different treatment groups are shown in Figure 7C–H. Given the outstanding antibacterial performance of Ti_3_C_2_+L+CHX treatment observed in vivo, we propose that this regimen could potentially hinder the progression of ECC. On Day 48, the harvested teeth exhibited morphological and color characteristics indicative of pit‐and‐fissure caries, as shown in Figure 7I, closely resembling the types of dental caries commonly found in humans. The Ti_3_C_2_+L+CHX group demonstrated significantly smaller caries lesion sizes and better‐preserved enamel surfaces than other treatment groups. These findings indicate that the Ti_3_C_2_+L+CHX group may represent a promising strategy for the management and prevention of ECC.

**Figure 8:**
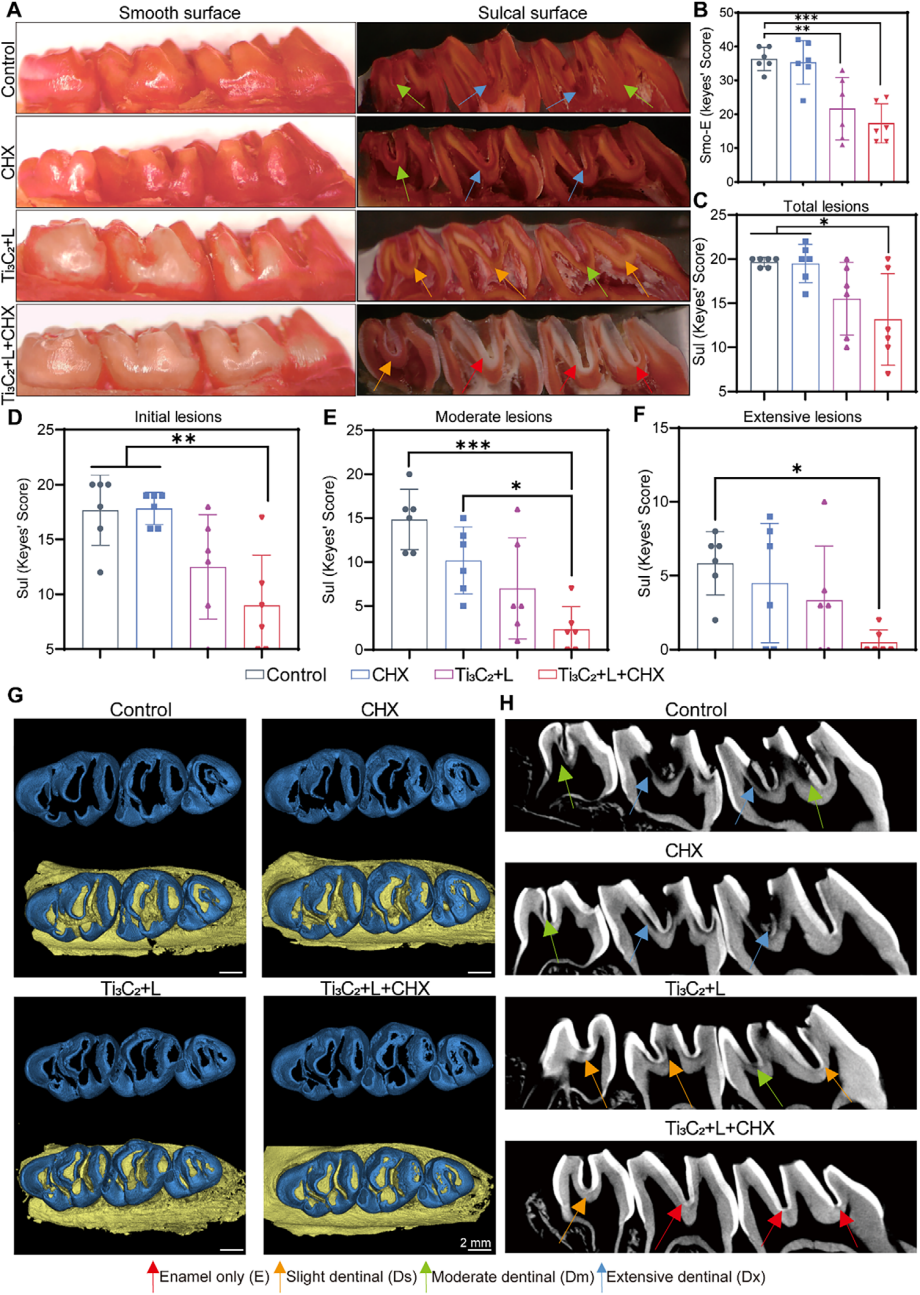
Anti‐ECC efficacy as evaluated by Keyes’ scoring and micro‐CT analysis. A) Representative images of carious lesions stained by murexide (red arrows, affected enamel; orange arrows, affected slight dentinal; green arrows, affected moderate dentinal; blue arrows, affected extensive dentinal; Control: PBS, CHX: treatment with CHX, Ti_3_C_2_+L: Ti_3_C_2_ upon laser irradiation, Ti_3_C_2_+L+CHX: Ti_3_C_2_ upon laser irradiation and CHX). B) Assessment of smooth‐surface lesions. C–F) Assessment of the sulcal surface lesions. G) Reconstructed images show the enamel loss on occlusal surfaces (scale bar, 2 mm). H) 2D sagittal scale images of the maxillary molars. The data are shown as mean ± SD with *n* = 6; ^*^ *p* < 0.05; ^**^ *p* < 0.01; ^***^ *p* < 0.001.

To precisely measure the targeted caries‐preventive efficacy of Ti_3_C_2_‐mediated PTT in combination with an antibacterial agent, we used Keyes' scoring method and micro‐CT analysis to assess the depth and extent of the carious lesions across various groups.^[^ ^51^ ^]^ As depicted in **Figure** **8**A, to delineate their areas and depths according to Keyes' scoring system, carious lesions were categorized into four distinct levels: enamel only (E), minor dentinal involvement (Ds, less than 1/4 of the dentin region), moderate dentinal involvement (Dm, 1/4 to 3/4 of the dentin region), and severe dentinal involvement (Dx, >3/4 of the dentin region).^[^ ^50^, ^52^ ^]^ Quantification revealed a significant reduction in the E scores on smooth surfaces in the Ti_3_C_2_+L+CHX group (Figure 8B). Furthermore, the severity of carious lesions on the sulcal surface can be categorized into four levels: total lesions (E+Ds+Dm+Dx), initial lesions (Ds+Dm+Dx), moderate lesions (Dm+Dx), and severe lesions (Dx). The prevalence and intensity of caries were markedly diminished in the Ti_3_C_2_+L+CHX group (Figure 8C–F). Micro‐CT analysis provides a direct approach for evaluating the anti‐caries effects of Ti_3_C_2_‐mediated PTT in tandem with antiseptic treatment in vivo.^[^ ^52^ ^]^ Figure 8G shows that the teeth in the Ti_3_C_2_+L+CHX group exhibited greater preservation of intact enamel (blue), which was detached and reconstructed from the maxillary molars. The corresponding sagittal slice image in Figure 8H shows that the high‐density regions in the Ti_3_C_2_+L+CHX group were relatively uniform and intact, with a limited number of low‐density demineralization sites and minimal demineralization severity, as indicated by the blue and green arrows. The Ti_3_C_2_+L group also exhibited a reduced number of low‐density demineralization sites. In contrast, the Control and Antibacterial agent groups displayed substantial low‐density shadows in the pit and groove regions, with numerous shadows extending into the medullary cavity in some instances.

### 2.8 In Vivo Safety Assessment of Ti_3_C_2_‐Mediated PTT

**Figure 9:**
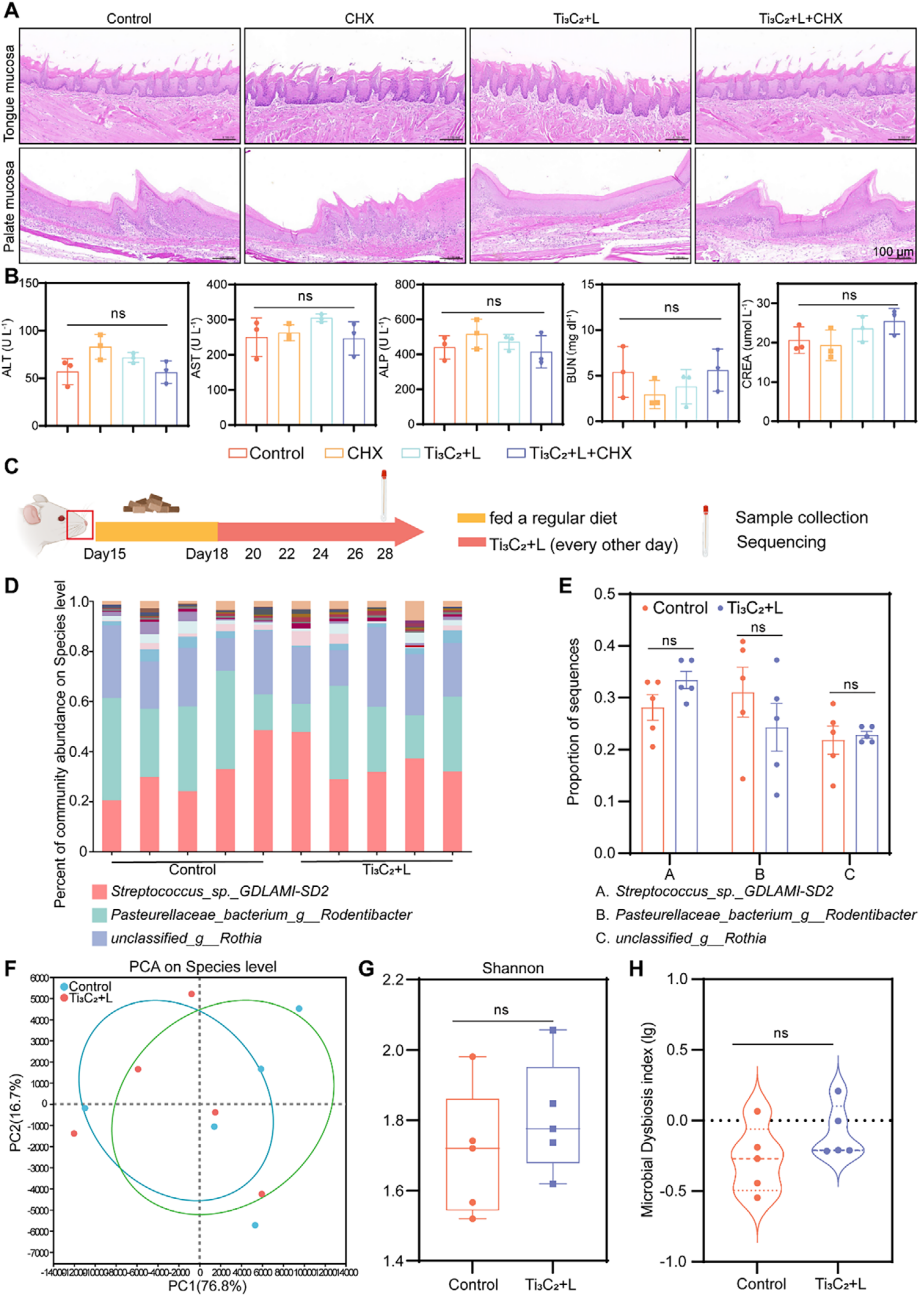
The biocompatibility of Ti_3_C_2_‐mediated PTT in vivo. A) H&E staining images for tongue mucosa and palate mucosa (scale bar, 100 µm). B) Blood biochemistry analysis, testing alanine aminotransferase (ALT), aspartate aminotransferase (AST), alkaline phosphatase (ALP), blood urea and nitrogen (BUN) and creatinine (CRET). C) Schedule of rat model construction for testing the oral microbiota homeostasis after Ti_3_C_2_ mediated PTT. D) Bar plot of microbial community composition in the Control and Ti_3_C_2_+L groups. E) Statistical testing for differences in dominant microbial populations between the Control and Ti_3_C_2_+L groups. F) PCA shows the contrast between the Control and the Ti_3_C_2_+L groups based on the features of microbial communities. G) Shannon index shows no noticeable differences between the Control and Ti_3_C_2_+L groups. H) MDI plot showing the inter‐group differences in microbial communities at the genus level between the Control and Ti_3_C_2_+L groups (ns, *p* > 0.05).

The biocompatibility of Ti_3_C_2_ was first confirmed by the Cell Counting Kit‐8 (CCK‐8) test. As shown in (Figure S18, Supporting Information), after co‐incubation with different concentrations of Ti_3_C_2_ in the dark for 24 h, no difference was observed in the survival rates of normal cells (mouse fibroblasts: L929; Mouse embryonic fibroblast cells: NIH3T3). We subsequently aimed to ascertain whether Ti_3_C_2_‐mediated PTT poses safety concerns in vivo. The in vivo biocompatibility of Ti_3_C_2_‐mediated PTT was evaluated through histological examination of the oral mucosa, major organs, and blood chemistry analysis. To confirm the safety of the treatment for tissues adjacent to the irradiated area, samples of the tongue and palate mucosa were obtained and subjected to hematoxylin and eosin (H&E) staining for assessment (**Figure** **9**A). Furthermore, blood biochemical tests were performed to evaluate the status of renal and liver functions (Figure 9B). The results showed no obvious differences between these groups. Throughout the treatment phase, there was a consistent rise in the body weight of the rats, with no significant differences noted among the four groups (Figure S19, Supporting Information). Finally, there were no abnormal changes in the histopathological structures of the major organs in all groups (Figure S20, Supporting Information).

The equilibrium of the indigenous oral microbiota is crucial for preventing the incursion of pathogenic bacteria and fostering oral health. Neonatal rats were subjected to Ti_3_C_2_‐mediated PTT without antiseptic administration or bacterial inoculation. Oral microbiota samples from the Control and Ti_3_C_2_‐mediated PTT groups were collected and analyzed for microbial diversity using 16S rRNA sequencing (Figure 9C). A bar chart displays the microbial composition and relative abundance of the two groups, with *Streptococcus*, *Rodentibacter*, and *Rothia* being the dominant bacterial genera (Figure 9D). Further differential testing of the abundance of dominant bacterial genera between the two groups revealed no statistically significant differences (Figure 9E). Principal component analysis (PCA) revealed a high degree of similarity between the microbiota of the two groups (Figure 9F), which was further confirmed by Shannon index analysis (Figure 9G), indicating no marked difference in microbial diversity between the two groups. Finally, the extent of microbial dysbiosis was quantified using the microbial dysbiosis index (MDI), in which a higher value indicated a greater degree of microbial imbalance. As shown in Figure 9H, no notable difference was observed in MDI between the Control and Ti_3_C_2_‐mediated PTT groups. This suggests that Ti_3_C_2_‐mediated PTT does not disrupt the integrity of the oral microbial community or induce any discernible dysbiosis. Regarding safety, Ti_3_C_2_ is known for its excellent biocompatibility,^[^ ^53^ ^]^ particularly the titanium element, which has a broad range of clinical applications in oral medicine. This implies that it does not cause adverse reactions in human tissues.^[^ ^54^ ^]^ Most importantly, our in vivo experiments and animal studies confirmed that Ti_3_C_2_‐mediated PTT is safe. It did not cause adverse effects on host tissues during the in vivo treatment, and 16S rRNA sequencing confirmed that the PTT effect of the material had no detrimental effect on the diversity of the original oral microbiota.

## 3 Conclusion

In conclusion, we uncovered an unforeseen role of the photothermal properties of Ti_3_C_2_ MXene in addressing persistent cariogenic biofilms and improving anti‐caries efficacy. This finding is particularly significant given that current treatments are inadequate for controlling bacterial infections and the severe progression of caries in patients with ECC. The application of the Ti_3_C_2_ MXene photothermal treatment effectively inhibited and eradicated persistent cariogenic bacteria, thereby addressing the adverse consequences associated with frequent antibiotic use, which can lead to the proliferation of resilient bacterial strains. Furthermore, the combined application of the Ti_3_C_2_ MXene photothermal treatment and antiseptic completely halted the progression of dental caries in rodent models. This therapeutic synergy has not been previously observed in this simulated ECC animal model. The improvements achieved through this combined approach can be attributed to two key factors: i) Ti_3_C_2_ upregulated HSP family members, promoting the depolymerization of misfolded proteins and inhibiting dormancy in persister under photothermal conditions, and ii) disrupt bacterial cell membranes, leading to increased permeability. The coordinated action of these mechanisms enhances resistance against persistent bacteria while slowing caries progression. These findings could aid in developing new strategies for enhancing ECC treatment.

## 4 Experimental Section

### Preparation of Ti_3_C_2_

Ti_3_AlC_2_ of 2 g was slowly added to 40% HF and stirred at 35 °C for 48 h. The mixture was washed with deionized water and centrifuged at 3500 rpm to remove the supernatant. This centrifugation process was repeated until the pH value of the solution reached 6–7, while retaining the sediment at the bottom of the centrifuge tube to obtain Ti_3_C_2_TX MXene. Subsequently, a solution of 40% (w/w) tetrabutylammonium hydroxide (TPAOH) was added to the sediment and stirred for an additional 48 h at room temperature. The excess base was removed by centrifugation at 15 000 rpm. The resulting black precipitate was dissolved in 40 mL of deionized water. The monolayer Ti_3_C_2_ MXene in the supernatant was collected by centrifugation at 12 000 rpm for 30 min. The product was then freeze‐dried under a vacuum and weighed for subsequent experiments.

### Material Characterization

A FE‐SEM (Zeiss, Germany) and TEM (JEOL‐2100F, Japan Electronics) were used to obtain morphology characterization. An AFM (Agilent 5500, Bruker Dimension Icon) was applied to measure the thickness of each nanosheet.

The ultraviolet‐visible (UV–vis) spectra were obtained using a UV–vis spectrophotometer with quartz cuvettes that had a path length of 10 mm. Photothermal images were recorded using a thermal imaging camera (Teledyne FLIR, USA). The X‐ray diffraction (XRD) pattern of the nanosheets was obtained using a Rigaku SmartLab SE (Japan) X‐ray diffractometer with a scanning angle range of 2 °–90 ° and a scanning rate of 2 ° min^−1^.

### Bacterial Strains and Culture Conditions

Three standard bacterial strains were utilized: *S. mutans* (UA159 & ATCC 700 610), *S. sobrinus* (ATCC 27 352), and *S. sanguinis* (SK36 & ATCC BAA‐1455D‐5). The optical density (OD) of the bacterial suspensions was adjusted to 1.4 at a wavelength of 600 nm. The bacterial plates were cultured in BHI (CM917, Beijing Land Bridge Technology) medium under microaerophilic conditions with 5% CO_2_ at 37 °C. For the animal experiments, dental plaques were collected using oral swabs and then dispersed in phosphate‐buffered saline (PBS, Servicebio, China) via sonication. Additionally, a selective medium, Mitis salivarius agar (MSB, Shandong Tuopu Biol‐Engineering Co., Ltd.) was used in this experiment, supplemented with bacitracin and potassium tellurite for the isolation of *Streptococcus* spp.

### Persistence Time‐Kill Curves and Growth Curves

Planktonic bacteria were separately cultured to the stationary phase, mixed with different concentrations of the antiseptic (MH), and incubated at 37 °C. Samples were taken at different time points for the time‐kill curve. Then, they were serially diluted and spread on agar plates, which were then incubated for 48 h before performing colony counts. For the growth curve, bacteria were co‐cultured with different concentrations of MH at 37 °C for 24 h. After centrifugation, they were resuspended in 1 mL of BHI and then added to fresh BHI for incubation at 37 °C, with samples taken at different time points for OD_600 nm_ measurements.

### Assessment of the Cariogenic Potential of Persisters

Bacteria that survived after treatment with a high concentration of MH were washed with PBS, centrifuged, resuspended in fresh BHI medium, and then cultured anaerobically at 37 °C for 24 h. Bacteria from different treatment groups were collected by centrifugation, and total RNA was extracted using a column‐based method (Vazyme Biotech, China). The purity and concentration of RNA from all samples were determined using a Nanodrop 2000 (Thermo Fisher, USA) before being converted to complementary DNA (cDNA) with the HiScript II Q RT SuperMix (Vazyme Biotech, China). The expression of cariogenic virulence‐related genes (*gtfB*, *gtfD*) was detected by quantitative real‐time polymerase chain reaction (qRT‐PCR), with primer sequences provided in Table S1 (Supporting Information). The persister bacteria were resuspended and cultured in a BHI medium containing 1% sucrose, and their biofilm formation ability was assessed through crystal violet staining.

### Assessment of Cell Membrane Permeability

Three types of bacteria were cultured to the stationary phase and then co‐cultured with MH at 40 MIC for 24 h to obtain persister cells. The bacteria were washed three times and resuspended in PBS. Propidium iodide (PI) was added to the control group and the cariogenic persister bacterial solutions to a final concentration of 10 µm, and these solutions were stored in the dark. The 100 µg mL^−1^ Ti_3_C_2_ was mixed with the persister suspension and then irradiated with an 808 nm laser for 5 min at a power density of 1.0 W cm^−2^. The supernatant was collected by centrifugation and transferred to a black 96‐well plate, and PI was added to a final concentration of 10 µm. The change in bacterial permeability after photothermal treatment with the material was measured. The fluorescence intensity of the samples was measured using a microplate reader at an emission wavelength of 615 nm and an excitation wavelength of 535 nm.

### Determination of HSP Expression

The expression levels of HSP were assessed using qRT‐PCR. RNA from the bacteria was extracted and reverse‐transcribed as described previously. The expression levels of HSP in mixed cariogenic bacteria were measured using qRT‐PCR with the primers listed in Table S1 (Supporting Information). The Cq values represent the cycle threshold in quantitative PCR. The relative expression levels of the target genes were calculated using the 2‐^ΔΔ^CT method, a widely accepted approach for determining the fold change in gene expression.

### Immunofluorescence Analyses

After treatment, biofilm‐coated slides were fixed with 4% paraformaldehyde for 30 min. Subsequently, the biofilm underwent lysozyme (5 µg mL^−1^) permeabilization, blocking, incubation with primary antibodies, and incubation with fluorescent secondary antibodies. Finally, the samples were observed and imaged using CLSM. The data were analyzed via Image J software.

### FISH Detection of Upregulated HSP Expression

Fluorescein‐labeled specific oligonucleotide probes (Servicebio, China) were used to bind specifically to target sequences, forming fluorescently labeled hybrids for the localization and quantification of target gene‐probe hybrids. After treatment, biofilm‐coated slides were fixed in situ hybridization fixative for 20 min, followed by three washes with PBS for 5 min each. The biofilms were then treated with Tris‐EDTA buffer containing 5 µg mL^−1^ lysozyme (Sigma, USA) for 10 min. Subsequently, pre‐hybridization solution was added, and the slides were incubated at 40 °C for 1 h. The pre‐hybridization solution was discarded, and probe‐containing hybridization solution was added, followed by overnight hybridization in a constant‐temperature incubator. After washing the hybridization solution, DAPI staining solution was added to the slides, and they were incubated in the dark for 8 min. After rinsing, an anti‐fade mounting medium was applied to seal the slides. The slides were observed, and 3D images were acquired using a Nikon confocal microscope (Nikon, Japan), with all steps performed in the dark. The probe sequences and specific excitation/emission wavelengths are provided in Table S2 (Supporting Information). The data were analyzed via Image J software.

### Collection and Confocal Measurement of PA

Bacteria from each treatment group were collected by centrifugation and resuspended in PBS. PA was extracted using the PROTEOSTAT Protein Aggregation Assay (Enzo, USA). Images were captured via CLSM (Zeiss, Germany), and Image J was used for fluorescence intensity analysis. In CLSM, the excitation wavelength was set at 550 nm, and PA was observed at the emission wavelength range of 600–620 nm. DAPI was used to observe nucleic acids.

### Nucleic acid Leakage Detection

The integrity of the bacterial cell membrane was assessed by measuring the amount of nucleic acids released by the bacteria. Collect cariogenic persistent flora before and after photothermal treatment. Samples were centrifuged at 6000 r·min^−1^ for 10 min at 4 °C. Following this, the supernatant was removed, and the absorbance was measured at OD_260 nm_.

### CCK8 Assay

Mouse fibroblast (L929) and embryonic fibroblast cells (NIH3T3) were cultivated in an incubator with a 5% CO_2_ atmosphere at a temperature of 37 °C, with the addition of 10% fetal bovine serum (Gibco, USA) and 1% penicillin/streptomycin. The in vitro cytotoxic effects of the Ti_3_C_2_ were evaluated using the CCK‐8 assay. L929 and NIH3T3 cells were plated in 96‐well plates at 10000 cells per well and incubated for 24 h. Subsequently, the cells were co‐cultured with various concentrations of Ti_3_C_2_ solutions (100, 80, and 60 µg mL^−1^) for 24 h.

### PTT of Ti_3_C_2_ Against Planktonic Persisters

The first model was designed to suppress the formation of persisters. A stationary phase bacterial suspension was incubated with a Ti_3_C_2_ for 4 h, followed by irradiation with an 808 nm laser (Laserwave, China) at a power density of 1.0 W cm^−2^ for 5 min. Subsequently, MH was added to achieve a final concentration of 10 times MIC. The second model aimed to eradicate mature persisters. Bacteria at the stationary phase were treated with MH at a final concentration of 40 times MIC for 24 h and incubated with the Ti_3_C_2_ for an additional 4 h. The bacterial suspension was subjected to laser irradiation under the same conditions as described above. Finally, the diluted bacterial suspension was plated onto BHI agar plates. After a 48‐h incubation, colony‐forming units (CFUs) were determined based on the dilution factors and the number of colonies formed by the monoclonal bacteria.

### Antimicrobial Effect on Multispecies Biofilm

Two distinct biofilm models were developed to evaluate the ability of Ti_3_C_2_‐mediated PTT to eradicate persisters in mature biofilms and inhibit their formation. Initially, equal concentrations and volumes of *S. mutans*, *S. sobrinus*, and *S. sanguinis* were combined in the BHI broth with 1% sucrose to create a mixture. For the biofilm eradication model, this bacterial mixture was added to wells and incubated for 24 h to allow biofilm formation. Subsequently, the biofilms were treated with 40 MIC of antiseptic (MH、CHX、CPC) for another 24 h. After washing with PBS, various concentrations of Ti_3_C_2_ (final concentrations of 60, 80, and 100 µg mL^−1^) were introduced into the wells. Following a 4‐h co‐incubation, the wells were exposed to laser irradiation for 5 min, as previously detailed. The effectiveness of biofilm eradication was then assessed using crystal violet staining, live/dead bacterial staining, and plating diluted samples on the BHI agar.

In addition, a separate model was constructed to assess the ability of Ti_3_C_2_‐mediated PTT to inhibit biofilm formation. In this model, multispecies bacterial mixtures (300 µL) and Ti_3_C_2_ (300 µL) were co‐incubated in a 24‐well plate for 4 h. The wells were irradiated with an 808 nm laser at an intensity of 1.0 W cm^−2^ for 5 min. After treatment, fresh BHI broth containing 1% sucrose was added, and the cultures were allowed to grow for an additional 20 h. Subsequently, a medium change was performed, and the cultures were incubated for another 24 h. Finally, the effectiveness of biofilm inhibition was determined using live/dead bacterial staining and plating diluted samples on BHI agar.

### Live/Dead Bacterial Staining

Live/dead bacterial staining was performed following the instructions provided by the Bacterial Viability Kit (LIVE/DEAD BacLight, Invitrogen, USA). To be specific, SYTO 9 (1.5 µL) and PI (1.5 µL) were each diluted in 1 mL of saline to prepare the staining solution. This solution, containing SYTO 9 and PI at their respective working concentrations, was applied to stain the biofilms for 15 min in the dark. After three rinses, the stained biofilms were covered with a mounting medium, and 3D images were captured along the z‐axis using CLSM, with each layer having a thickness of 0.5 µm. SYTO 9 dye was used for live bacteria staining, with excitation/emission wavelengths of 485/498 nm. The PI dye was used for dead bacteria staining, with excitation/emission wavelengths of 535/617 nm.

### Changes in Bacterial Morphology Following PTT

After the bacteria underwent different treatments, they were collected and fixed overnight at 4 °C using a fixative solution. Subsequently, the samples were rinsed, dehydrated, infiltrated, dried, and sputter‐coated with gold. Observations of the samples were then conducted using SEM and TEM techniques.

### Molecular Dynamics Simulation Method

The study employed MD simulations to describe the interactions between phospholipid membranes and nanoparticles. The composition of the simulation systems is listed in Table S3 (Supporting Information). All simulations were conducted using the Gromacs‐2020.7 software package, with the molecular force field determined by GAFF2. Finally, visualization software was applied for analysis.

### Intracellular ATP Detection

Three cariogenic bacterial strains were individually cultured to the stationary phase, centrifuged to discard supernatants, and then treated with 40 MIC of MH (final concentration) in triplicate wells per group. After 24 h incubation to obtain persister cells, the bacterial suspensions were centrifuged to remove supernatants. The persister cells were resuspended in 100 µL culture medium and co‐incubated with 100 µL Ti_3_C_2_ (final concentration 100 µg mL^−1^) for 4 h, followed by 808 nm laser irradiation for 5 min. Post‐treatment, the bacterial suspensions were centrifuged to remove supernatants, yielding persister populations before and after PTT. The harvested bacteria were resuspended in 100 µL PBS and transferred to a black 96‐well plate. After temperature equilibration, 100 µL BacTiter‐GloTM Reagent (Beyotime Biotechnology, China) was added to each well, followed by 15‐min incubation at room temperature protected from light. Chemiluminescence values were subsequently measured using a multifunctional microplate reader. For bacterial quantification, persister cells before and after Ti_3_C_2_ photothermal treatment were serially diluted and plated for CFU counting. The relative ATP content per bacterial cell was calculated by dividing the enzymatic data (from the microplate reader) by the total bacterial count in each group.

### In Vivo Experiment

The ethics of the animal experiments were approved, supervised, and supported by the Experimental Animal Center of Zhengzhou University (LAC20240906). Sprague‐Dawley rats, aged 15 days, were raised in the Animal Experiment Center at Zhengzhou University. Initially, to minimize the impact of indigenous oral flora, 15‐day‐old rats were fed with water containing benzylpenicillin and streptomycin sulfate, while their feed was supplemented with an appropriate concentration of antibiotics for a period of three consecutive days. Then, cotton swabs were used to smear the occlusal surface of the teeth for sampling, dispersed in 500 µL of PBS, and ultrasonicated for 3 min, followed by dilution and plating. The oral microbiota of the rats was verified to be at the same level by CFU. Three cariogenic bacteria were cultured to an OD_600 nm_ of 0.8 and mixed in equal volumes to prepare a mixed bacterial suspension. The mixed cariogenic bacterial suspension of 200 µL was applied twice daily to the bilateral maxillary molars of rats aged 18 to 21 days for four consecutive days to ensure effective bacterial colonization. Afterward, oral plaque was collected from the rats using an oropharyngeal swab, which was then plated onto MSB agar (containing 0.2 U mL^−1^ bacitracin and 0.01 mg mL^−1^ potassium tellurite) to verify the success of the bacterial inoculation.^[^ ^52^ ^]^ All rats were provided with a cariogenic diet known as 2000 (Jiangsu Xie Tong Pharmaceutical Bioengineering Co., Ltd.) and had access to 5% sucrose water throughout the duration of this experiment, starting from the age of 18 days.

Rats were categorized into four groups: Ti_3_C_2_+L+CHX, Ti_3_C_2_+L, CHX, and Control (*n* = 6 per group). The treatment commenced when the rats reached 21 days of age. Following anesthesia, Ti_3_C_2_ (3 mg mL^−1^), CHX (1 µm), or PBS was applied to the teeth of the rats. Subsequently, each side of the maxillary region's teeth was irradiated with an 808 nm laser for 3 min per session at a power density of 1.0 W cm^−2^. PTT was applied daily for four consecutive days, followed by treatments every two days until the rats reached 48 days of age. Food and water were withheld for at least 30 min following PTT administration. In the irradiation cycle, plaque samples were collected and cultured in the same manner as previously described on the 18th day, 21st day, 24th day, 30th day, 36th day, 42th day, and 48th day. The body weights of rats in each group were recorded.

### In Vivo Evaluation of Anti‐Caries Efficacy

Samples were collected and cariogenic bacteria were counted at various time points according to previously established methods. On the 48th day, all rats were euthanized via CO_2_ asphyxiation, and their maxillary teeth were extracted and photographed using a stereoscopic microscope. The severity of dental caries was assessed using micro‐CT (NEMO, China) and the Keyes' scoring system after staining with murexide (60 mg mL^−1^).

### In Vivo Safety Evaluation

Biological safety was assessed by collecting blood, heart, liver, spleen, lung, and kidney samples for blood biochemistry analysis and H&E staining. Sprague‐Dawley rats 21 days old were categorized into two groups, Ti_3_C_2_+L and Control, with five rats in each group. Post‐anesthesia treatment was performed as described above: once every two days. After four anesthesia sessions, oral and throat swabs were collected for 16S rRNA sequencing.

### Data Analysis

Data from at least three samples (*n* ≥ 3) are presented as mean ± standard deviation (SD). For statistical comparisons, a two‐tailed independent Student's *t*‐test was used to evaluate the differences between the two groups, and a one‐way analysis of variance (ANOVA) was utilized for the evaluation of differences among different groups. Significance levels were set at ^*^ *p* < 0.05, ^**^ *p* < 0.01, and ^***^ *p* < 0.001. A *p*‐value of <0.05 indicates statistical significance. Statistical analyses were performed using GraphPad Prism software (GraphPad Software, USA).

## Conflict of Interest

The authors have no conflicts of interest to declare.

## Author Contributions

Y.Y.Z. and L.L.Y. contributed equally to this work. Y.Y.Z. performed the experiments, processed the data, and wrote the manuscript. L.L.Y. contributed to the design of the study and discussed the results. J.J. and W.S.L. provided suggestions on the project. S.L., X.L.Z., and H.Y.Q. contributed to the animal experiments. D.F.L. provided suggestions and revised the article. R.L guided the experiments and technical support on the project.

## Supporting information



Supporting Information

## Data Availability

The data that support the findings of this study are openly available in Yinyin Zhang at https://doi.org/[doi], reference number 52.
