# Supplementary material for: A MXene‐Based Nanothermal Knife Inhibits Aggresome‐Mediated Persister Formation for Preventing Dental Caries
Source: Adv Sci (Weinh). 2025 Jun 10;12(33):e01501. doi: 10.1002/advs.202501501 (PMC12412528; doi:10.1002/advs.202501501)
Supplement: Supplementary file 1 — Supporting Information [file ADVS-12-e01501-s001.doc]

| Supporting Information  **A MXene-based nanothermal knife inhibits aggresome-mediated persister formation for preventing dental caries**  Yinyin Zhang^1a^, Leilei Yang^1a^, Jing Jiao^a^, Wenshuai Li^a^, Sen Lin^a^, Xianlong Zong^a^, Haoyang Qin^a^, Danfeng Liu^*a^, Rui Li^*a^  ^a^Department of Stomatology, The First Affiliated Hospital of Zhengzhou University, Zhengzhou 45000, China.  ^*^Corresponding Author: Email: [fcclir@zzu.edu.cn](mailto:fcclir@zzu.edu.cn); [liudanfeng@zzu.edu.cn](../../../1111改改改/ZYY/liudanfeng@zzu.edu.cn)  ^1^ Yinyin Zhang and Leilei Yang contributed equally to this paper  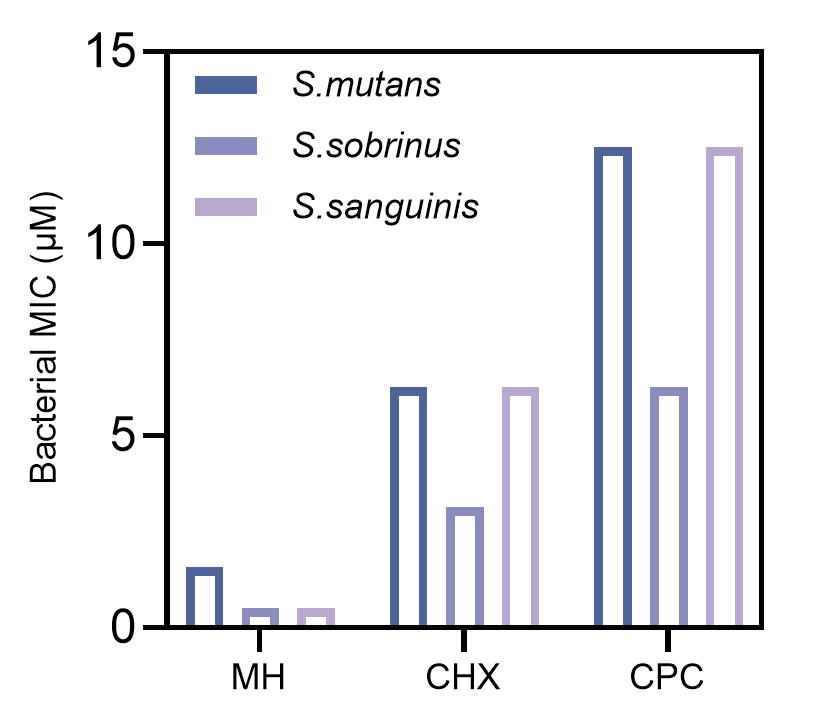  **Figure S1.** The minimum inhibitory concentrations (MIC) of Minocycline hydrochloride (MH), chlorhexidine (CHX), and cetylpyridinium chloride (CPC) against the cariogenic bacteria.  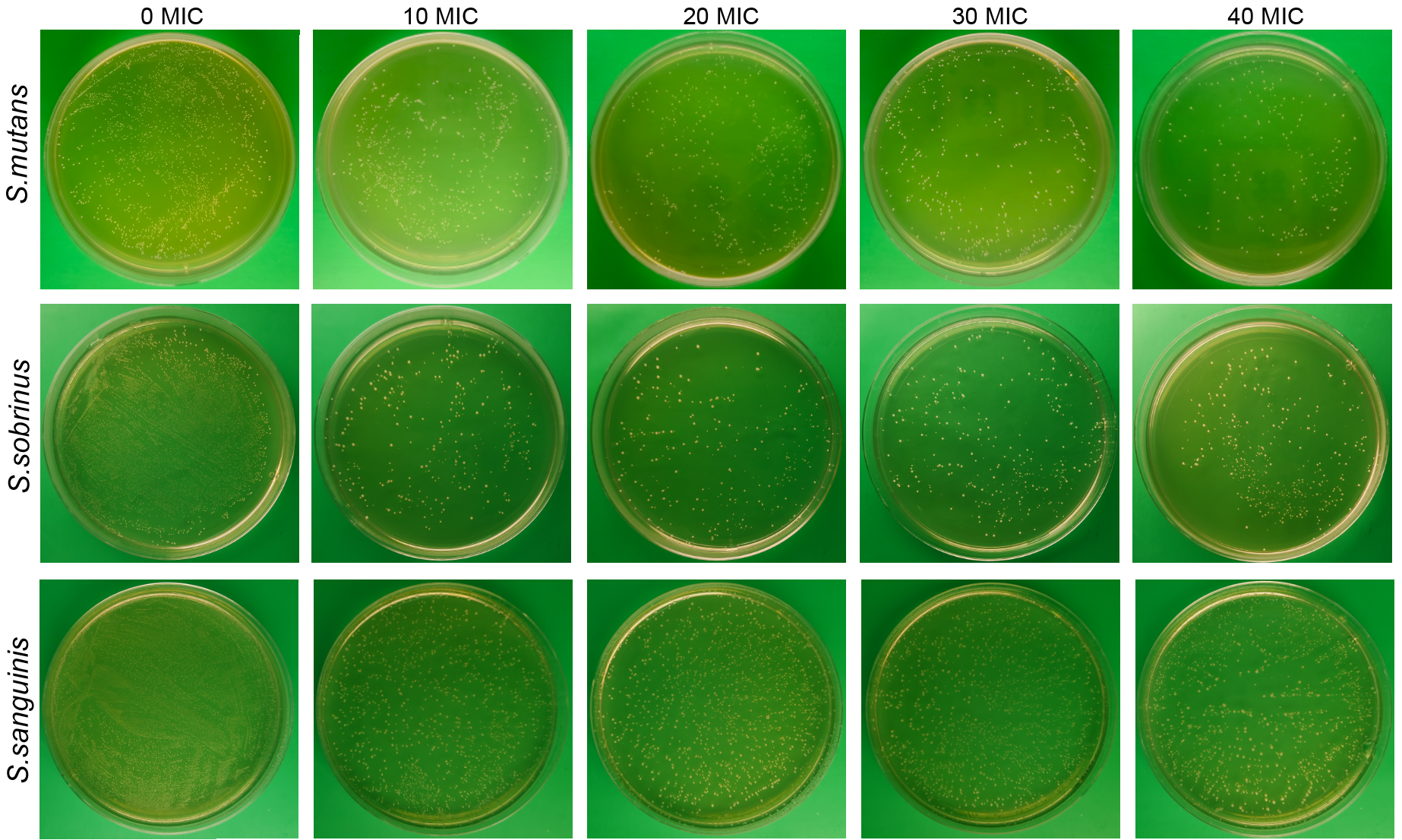  **Figure S2.** Representative images of bacterial colonies in antiseptic (MH) treatment group with different dilution factors for three bacterial species.  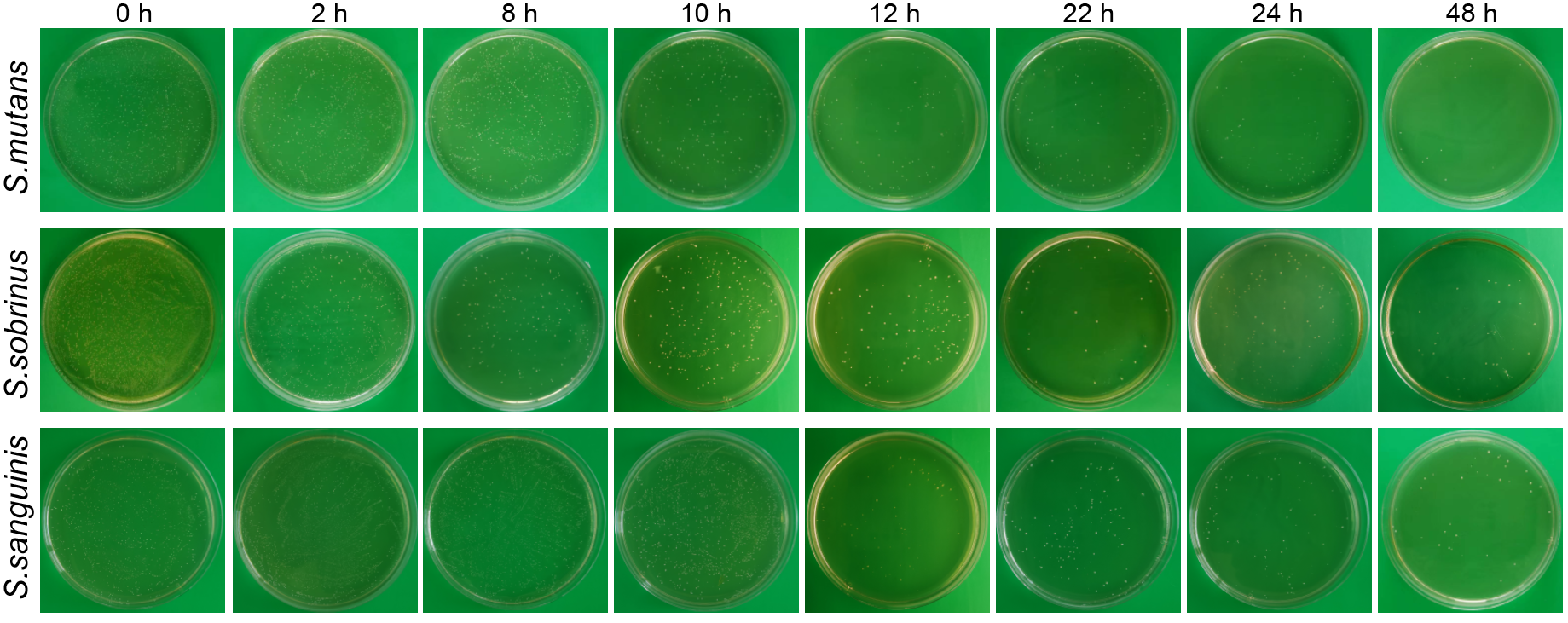  **Figure S3.** Representative pictures of the double-kill curves of three bacterial species.  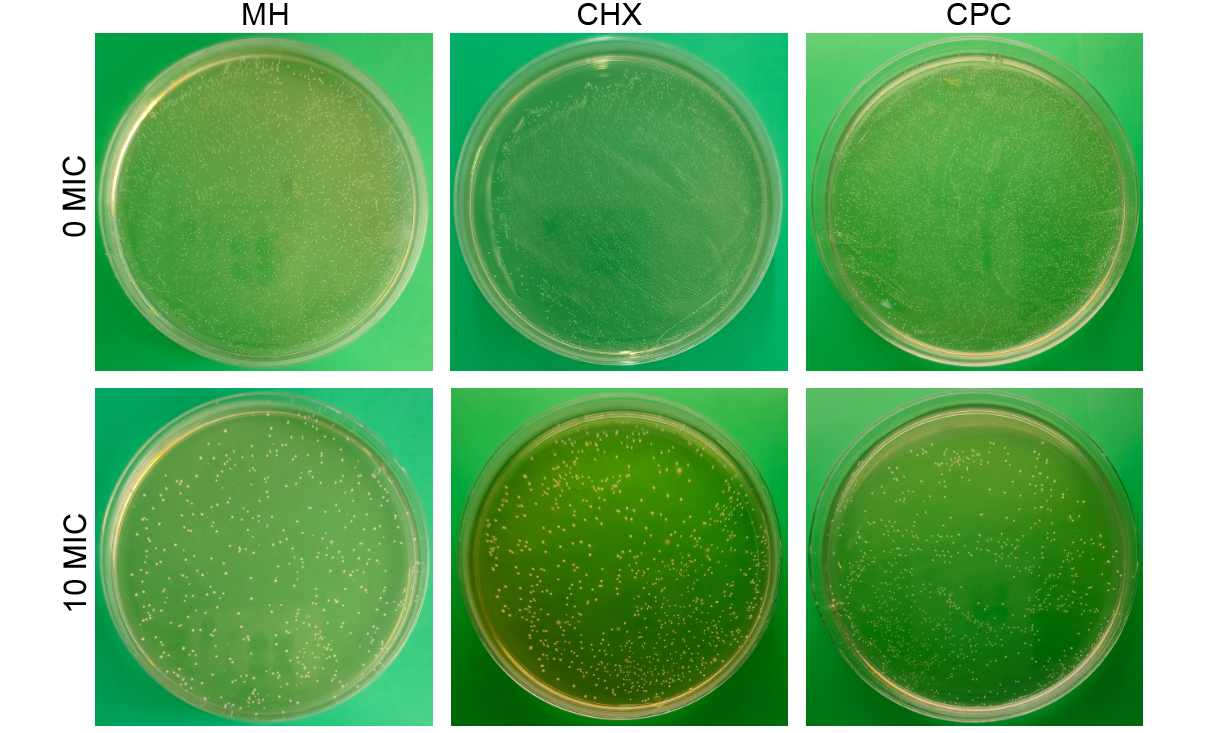  **Figure S4.** Representative images of multispecies biofilm subjected to different concentrations of antiseptics.  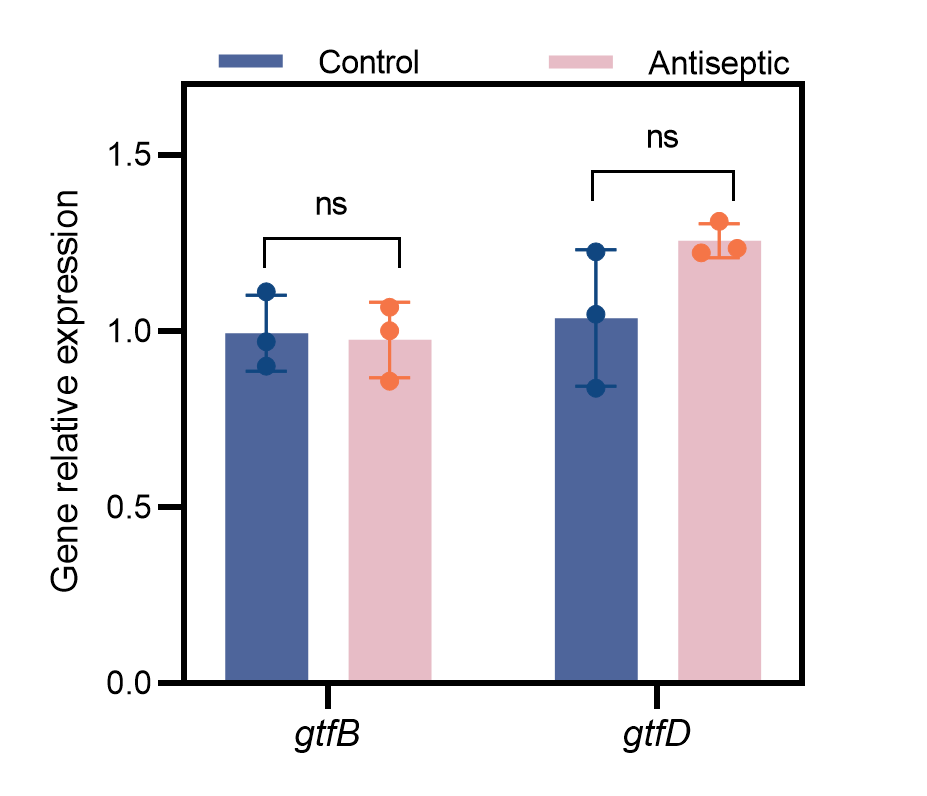  **Figure S5.** qPCR was employed to assess the expression levels of cariogenic virulence genes *gtfB* and *gtfD* (Antiseptic: MH; ns, *p* > 0.05)  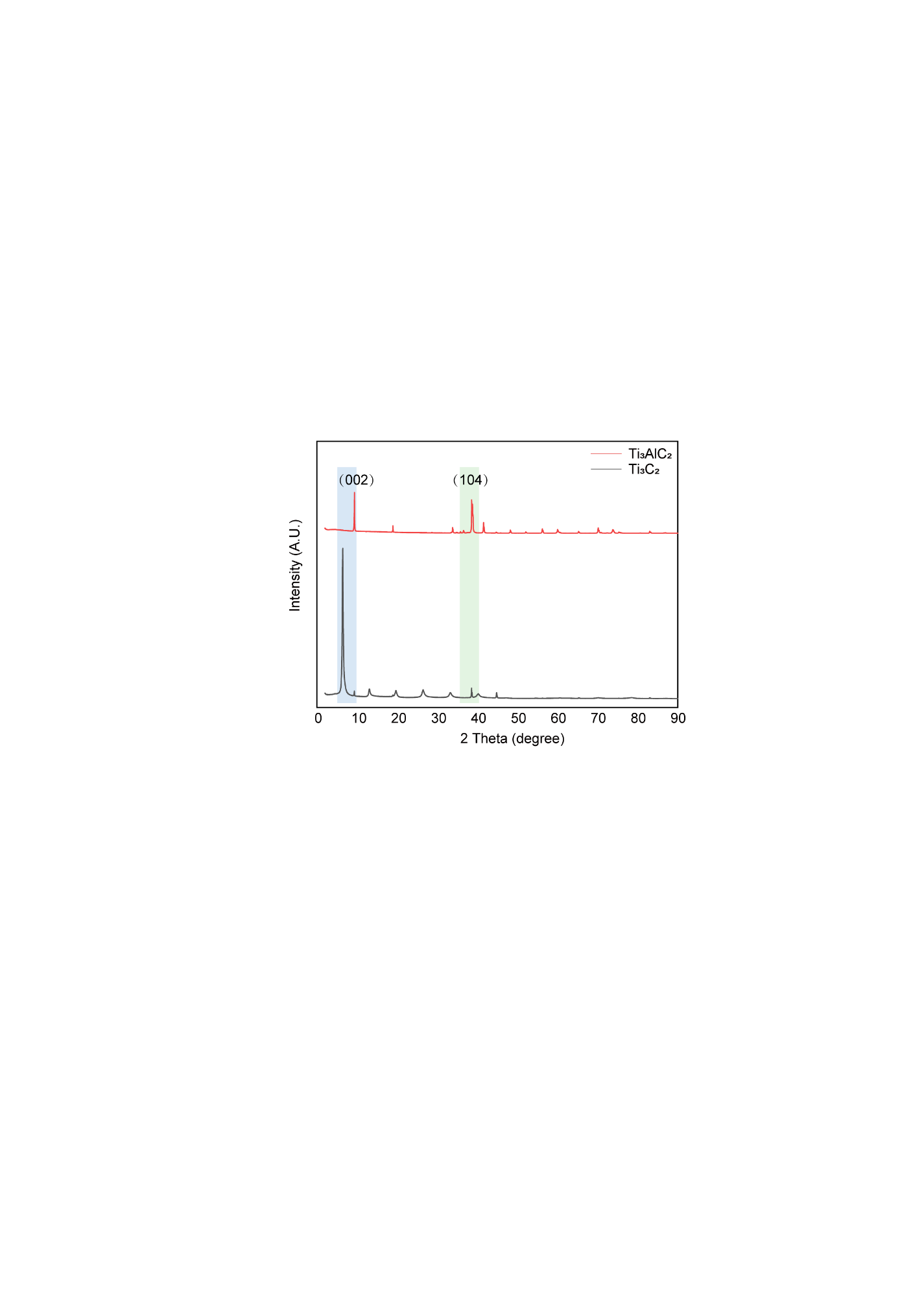  **Figure S6.** XRD spectra of Ti_3_AlC_2_ and Ti_3_C_2_. The characteristic peak at (104) was significantly attenuated in Ti_3_C_2_. The (002) diffraction peak of Ti_3_C_2_ was significantly shifted to the left.  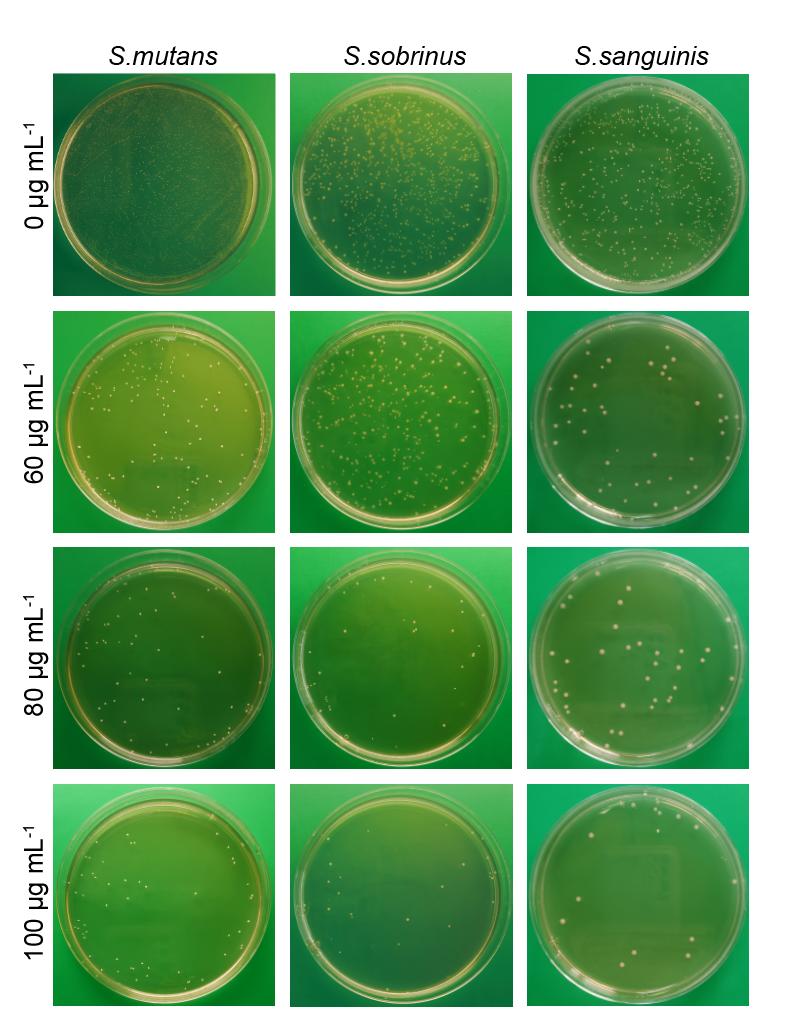  **Figure S7.** Representative images of Ti_3_C_2_-mediated PTT inhibition of persistent bacteria.  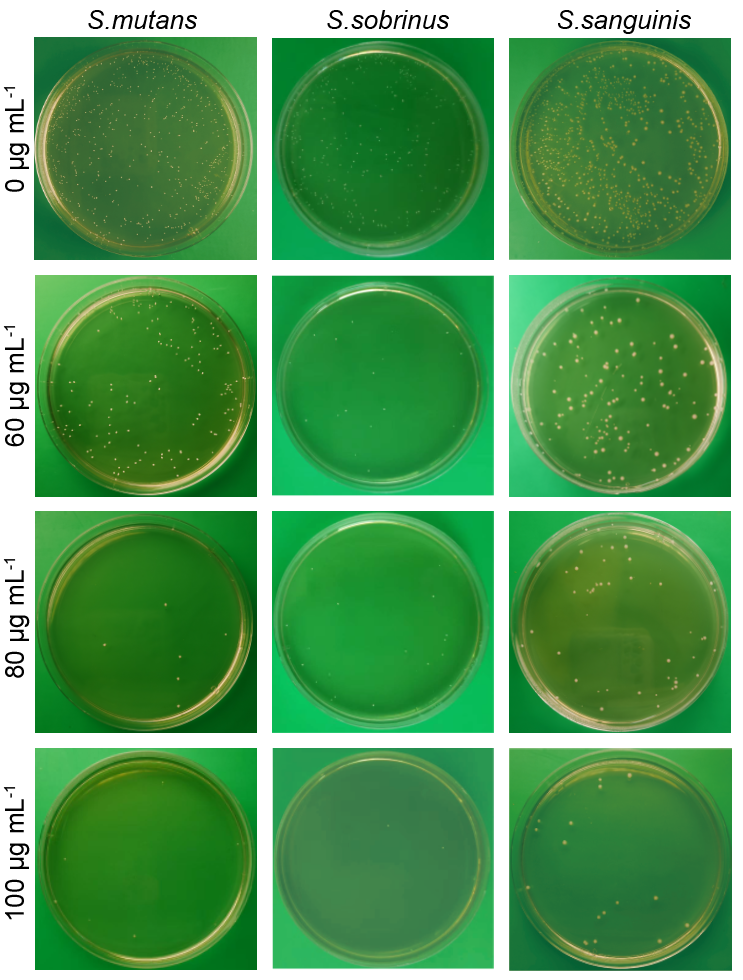  **Figure S8.** Representative images of Ti_3_C_2_-mediated PTT killing of mature persisters.  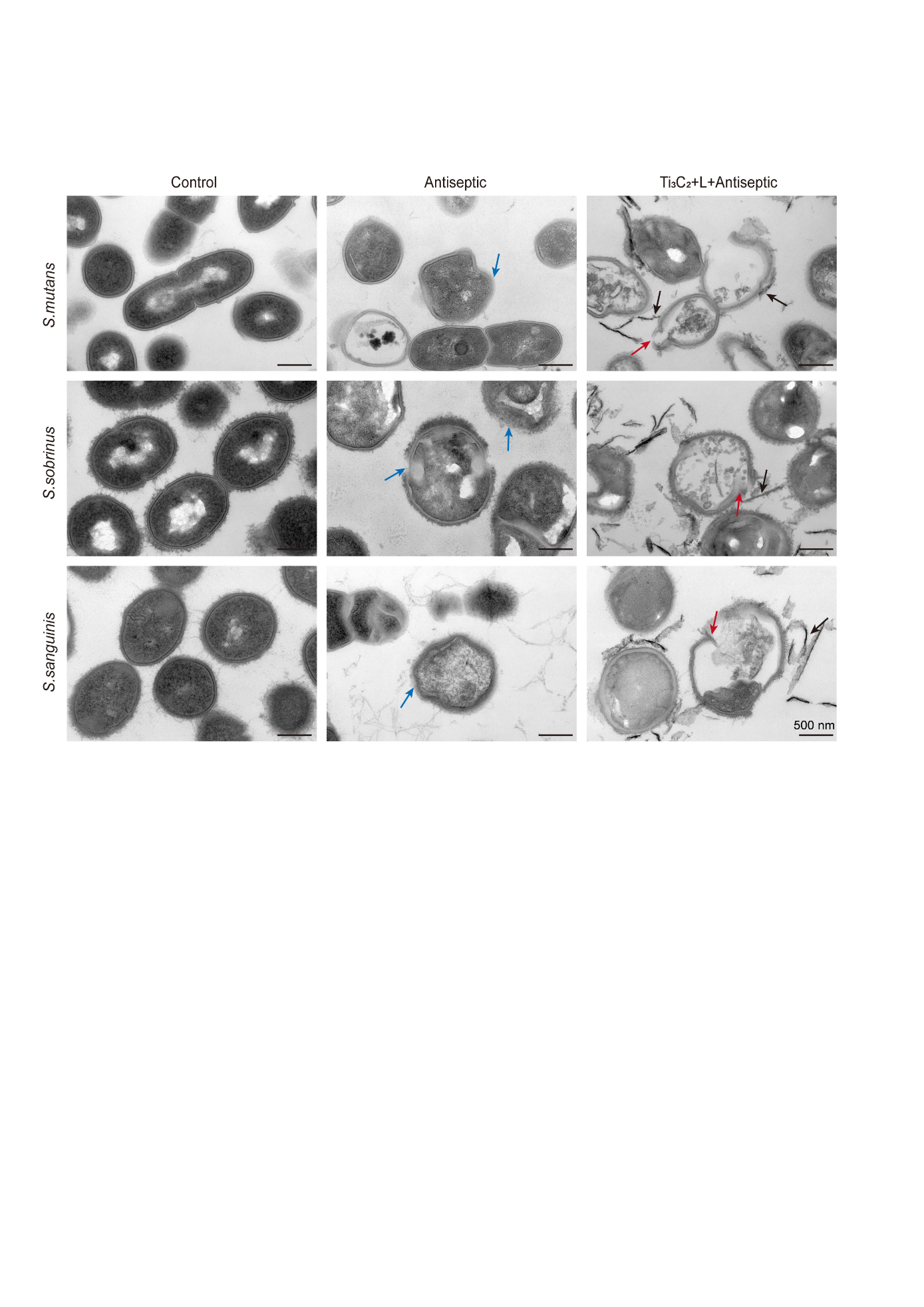  **Figure S9.** Morphological changes in bacteria treated with different treatment by TEM (Control: BHI; Antiseptic: treatment with 40 MIC of MH; Ti_3_C_2_+L+Antiseptic: treatment with 40 MIC of MH and 100 μg mL^-1^ Ti_3_C_2_ upon laser irradiation. Blue arrows indicate the bacterial cell wall structure is ill-defined, red arrows indicate the destruction of the bacterial structure; framed areas are enlarged on the right; scale bar, 500 nm).  **A**  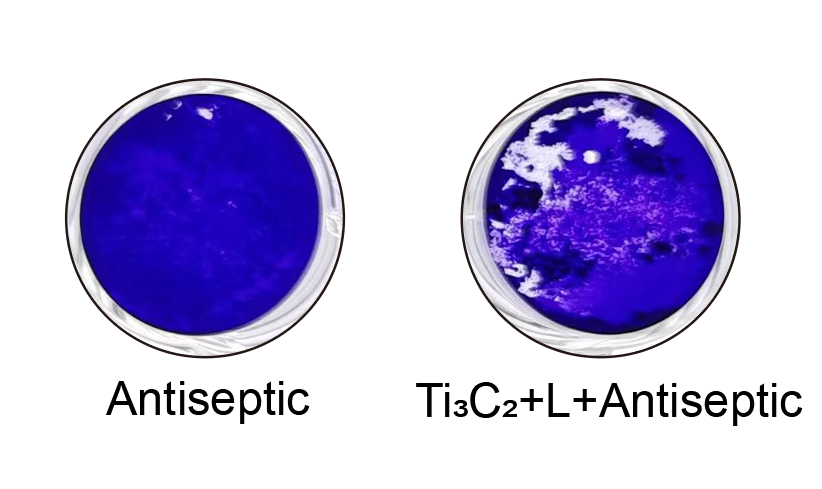  **B**  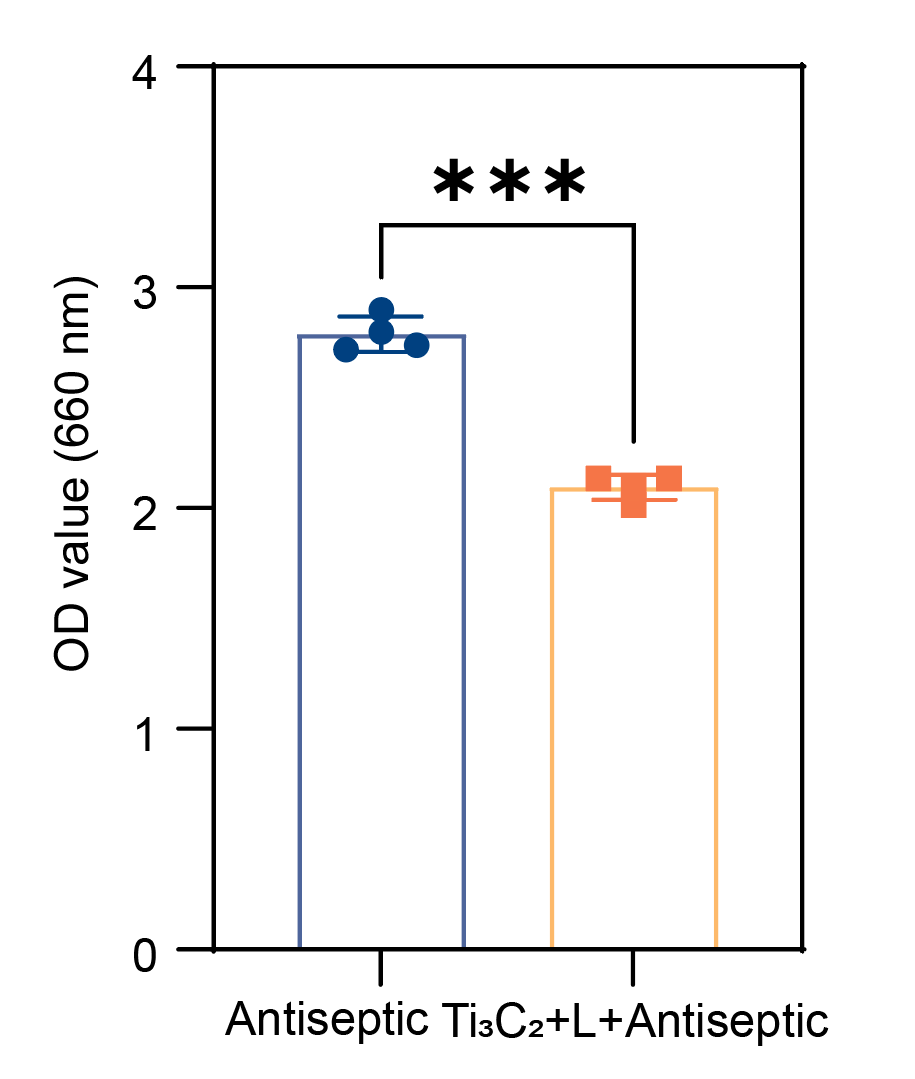  **Figure S10.** A) Representative images of crystal violet staining of multispecies biofilm after different treatments in biofilm eradication test (Antiseptic, treatment with 40 MIC of MH; Antiseptic+Ti_3_C_2_+L, treatment with 40 MIC of MH and Ti_3_C_2_ upon laser irradiation. *** *p* <0.001). and (B) Statistical analysis.  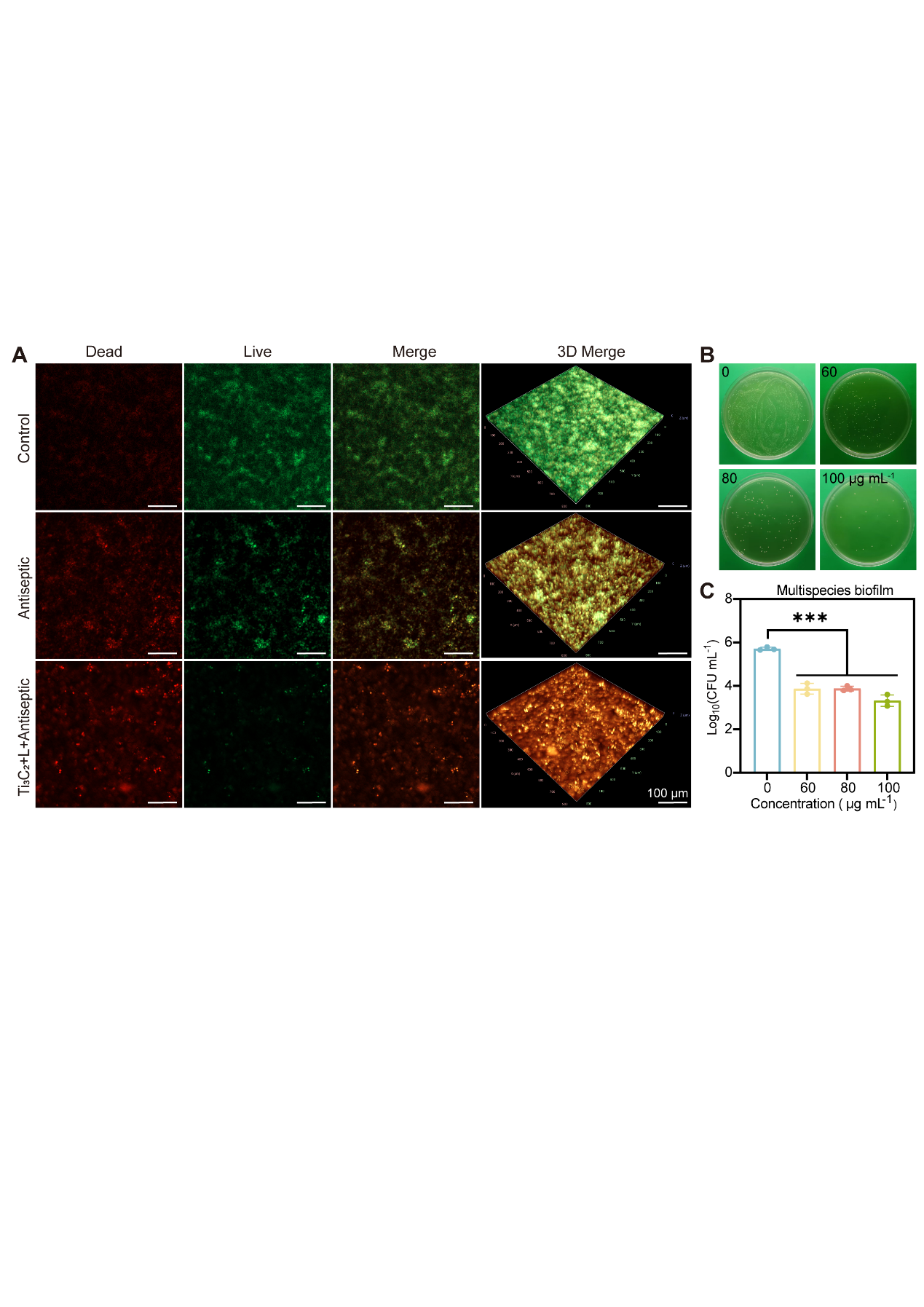  **Figure S11. Antibiofilm efficiency of Ti_3_C_2_-mediated PTT in vitro.** A) Live/dead bacterial staining of multispecies biofilm following various interventions for the eradication assessment (scale bar, 100 μm; green, live bacteria; red, dead bacteria). B) Representative images of bacterial colonies in the Antiseptic and Ti_3_C_2_+L+Antiseptic groups. C) CFU counts for mixed multi-species biofilm eradication assays (Control: BHI, Antiseptic: 10 MIC of CHX, Ti_3_C_2_+L+Antiseptic: 10 MIC of CHX and Ti_3_C_2_ upon laser irradiation).  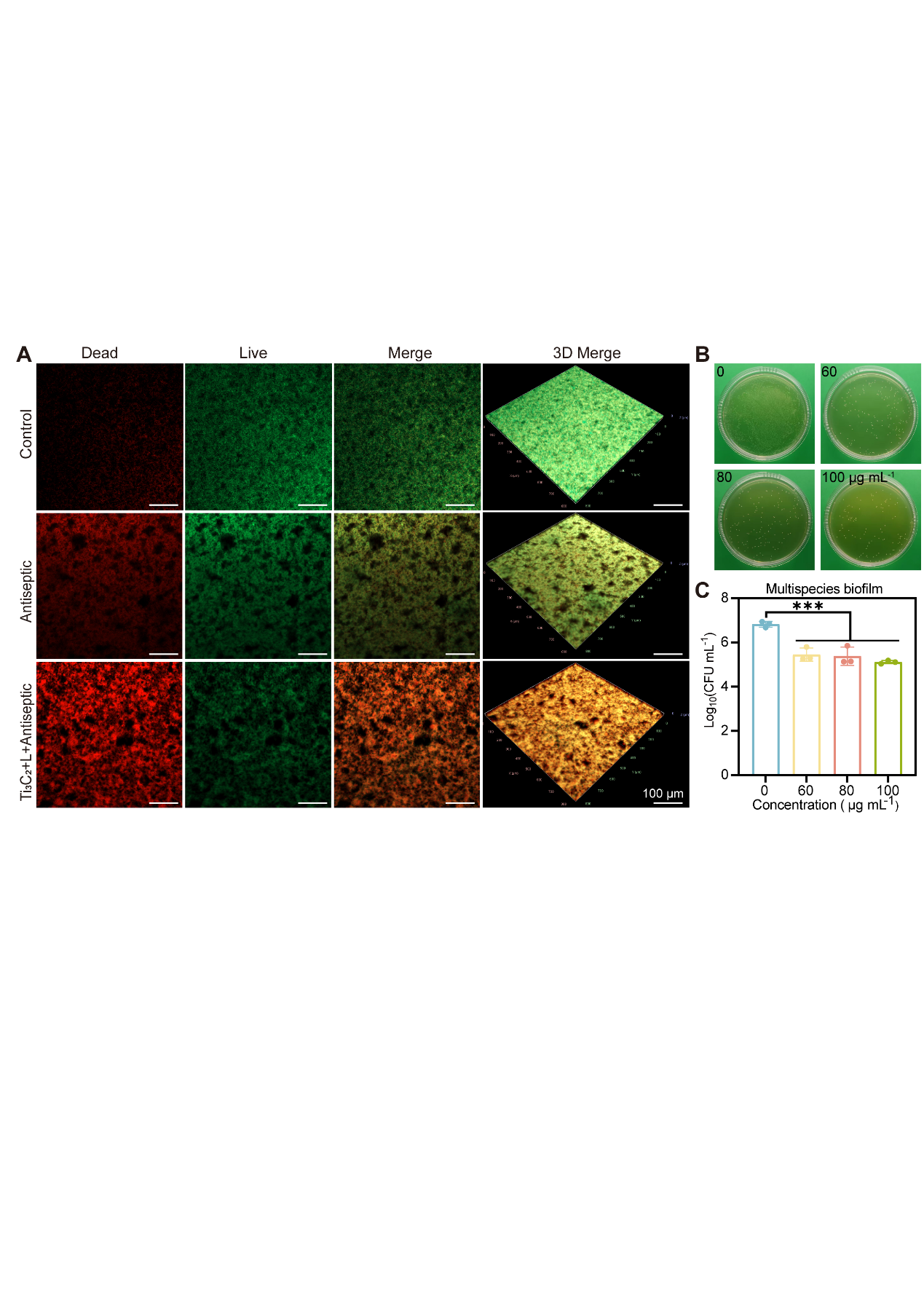  **Figure S12. Antibiofilm efficiency of Ti_3_C_2_-mediated PTT in vitro.** A) Live/dead bacterial staining of multispecies biofilm following various interventions for the eradication assessment (scale bar, 100 μm; green, live bacteria; red, dead bacteria). B) Representative images of bacterial colonies in the Antiseptic and Ti_3_C_2_+L+ Antiseptic groups. C) CFU counts for mixed multi-species biofilm eradication assays (Control: BHI, Antiseptic: 10 MIC of CPC, Ti_3_C_2_+L+ Antiseptic: 10 MIC of CPC and Ti_3_C_2_ upon laser irradiation).  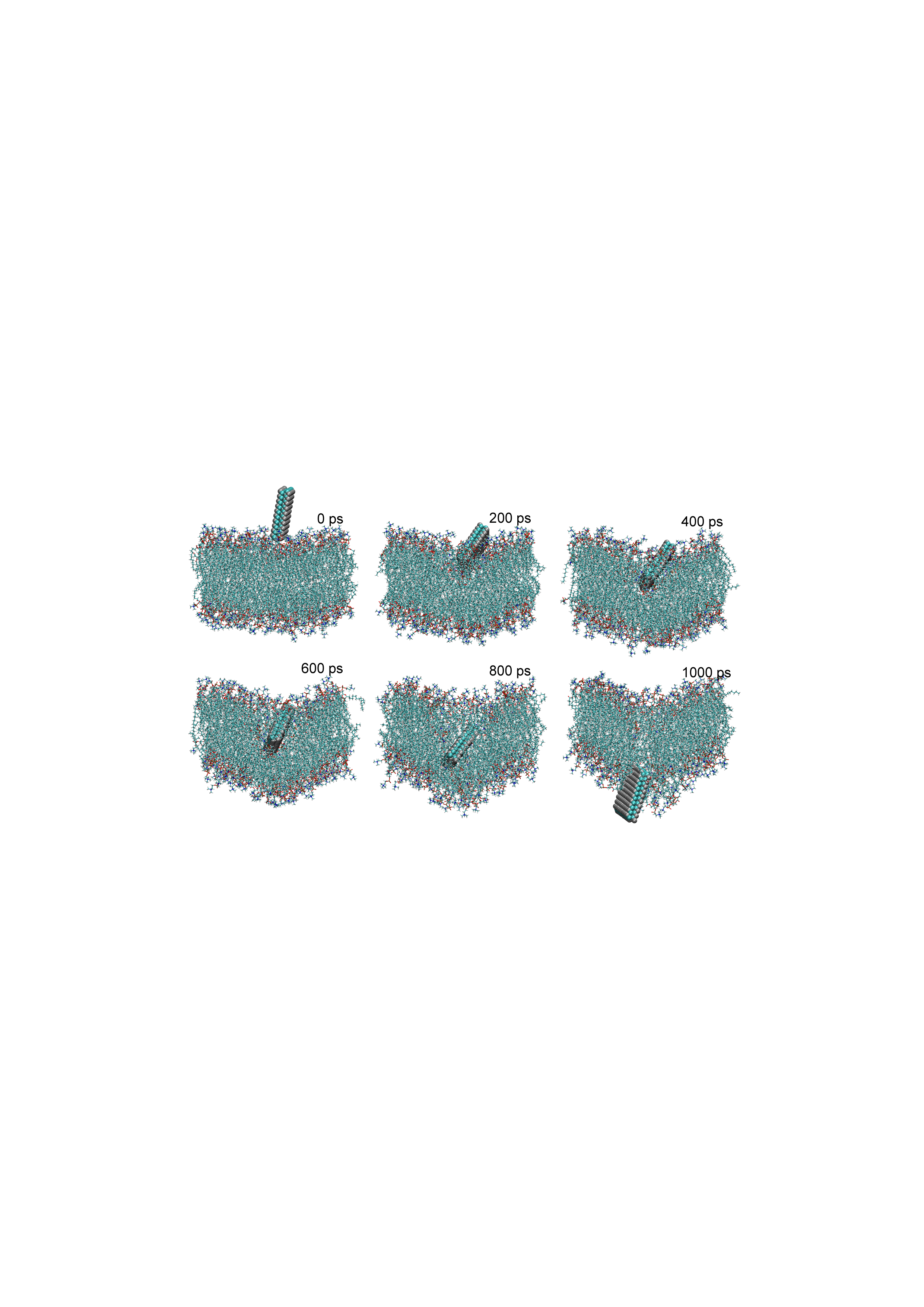  **Figure S13.** Time-dependent evolution of mass center distance between Ti_3_C_2_ surface and the lipid membrane (inset representative snapshots depicting the penetration processes; tempeture:55 ℃).  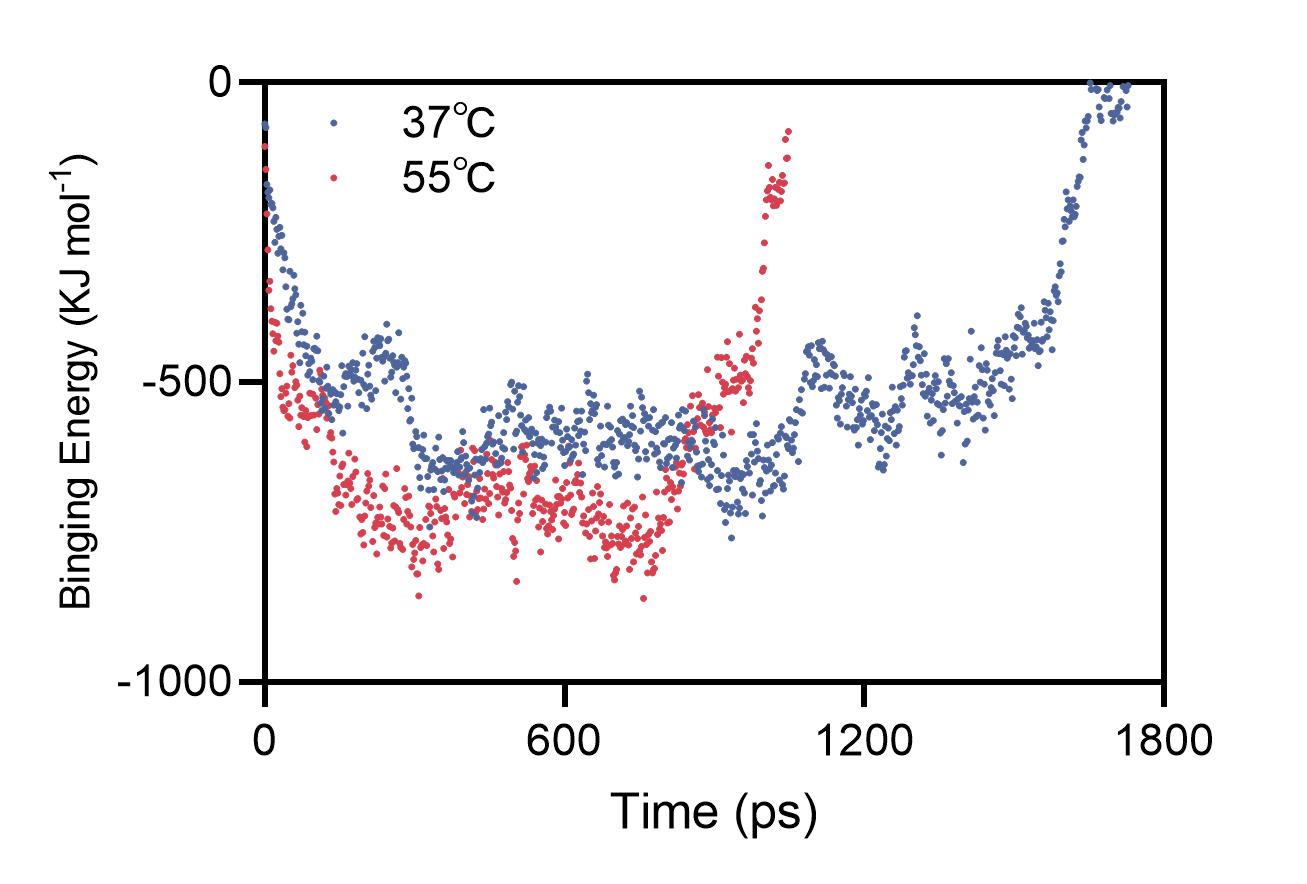  **Figure S14.** Interaction energy between Ti_3_C_2_ surface and the lipid membrane (inset: representative snapshots depicting the penetration processes).  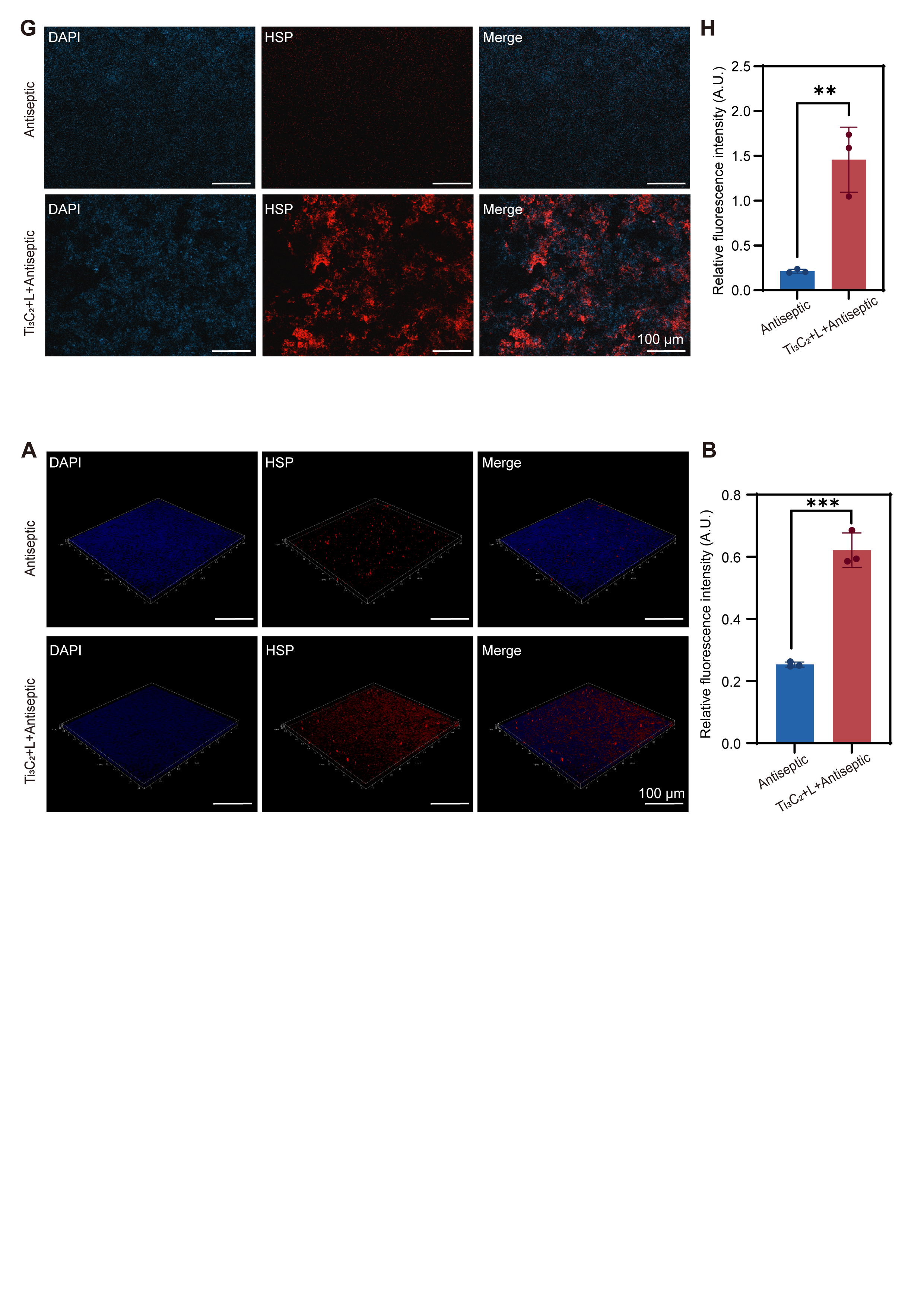  **Figure S15** The upregulation of HSP expression was detected using FISH analysis. A) Representative images of HSP expression and B) relative fluorescence intensity (Blue: DAPI; Red: HSP; scale bar, 100 μm). The data are shown as mean ± SD with n = 3; ***p* < 0.01, ****p* < 0.001.  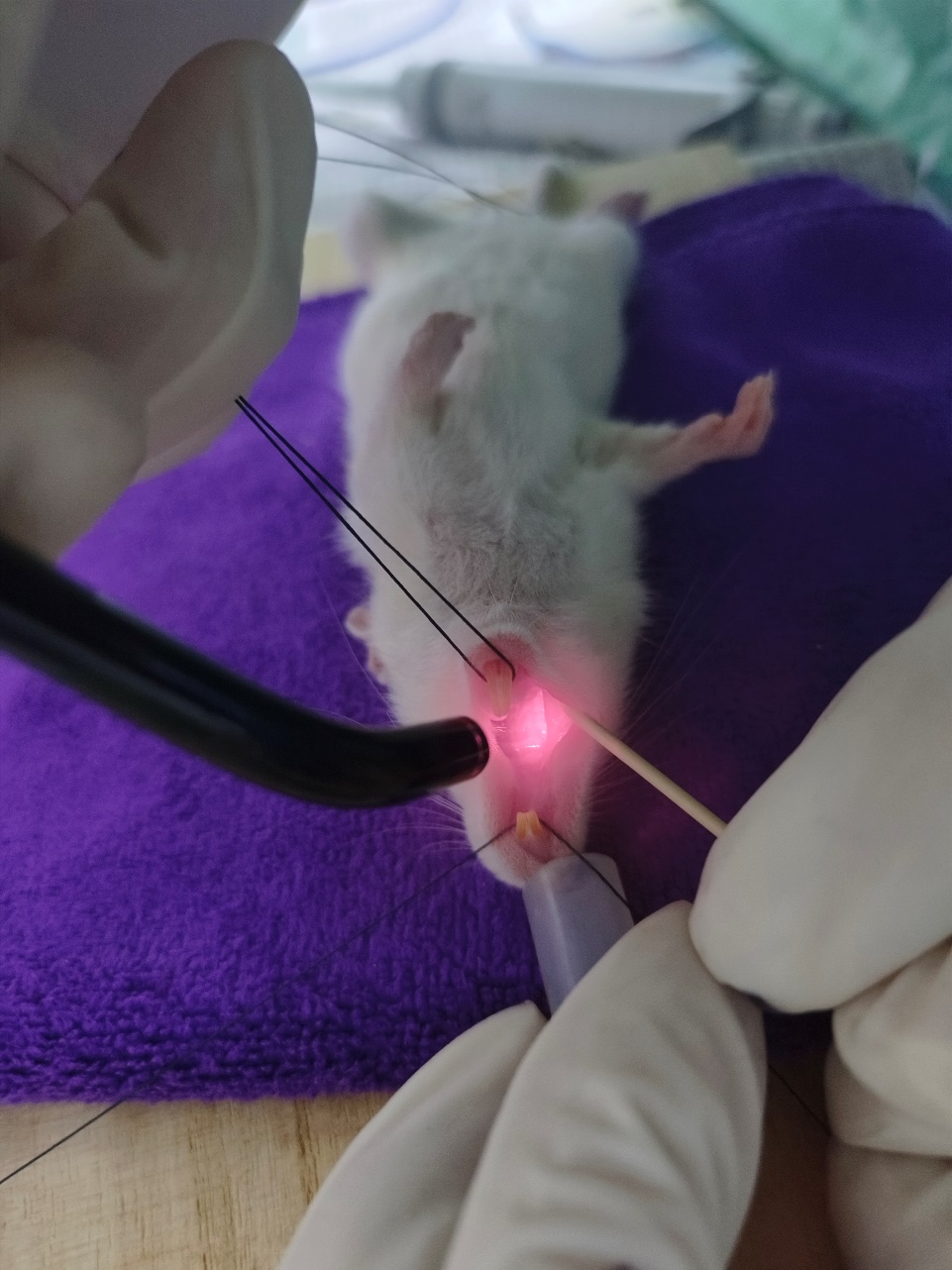  **Figure S16.** Sampling process via oral swabbing for rat.  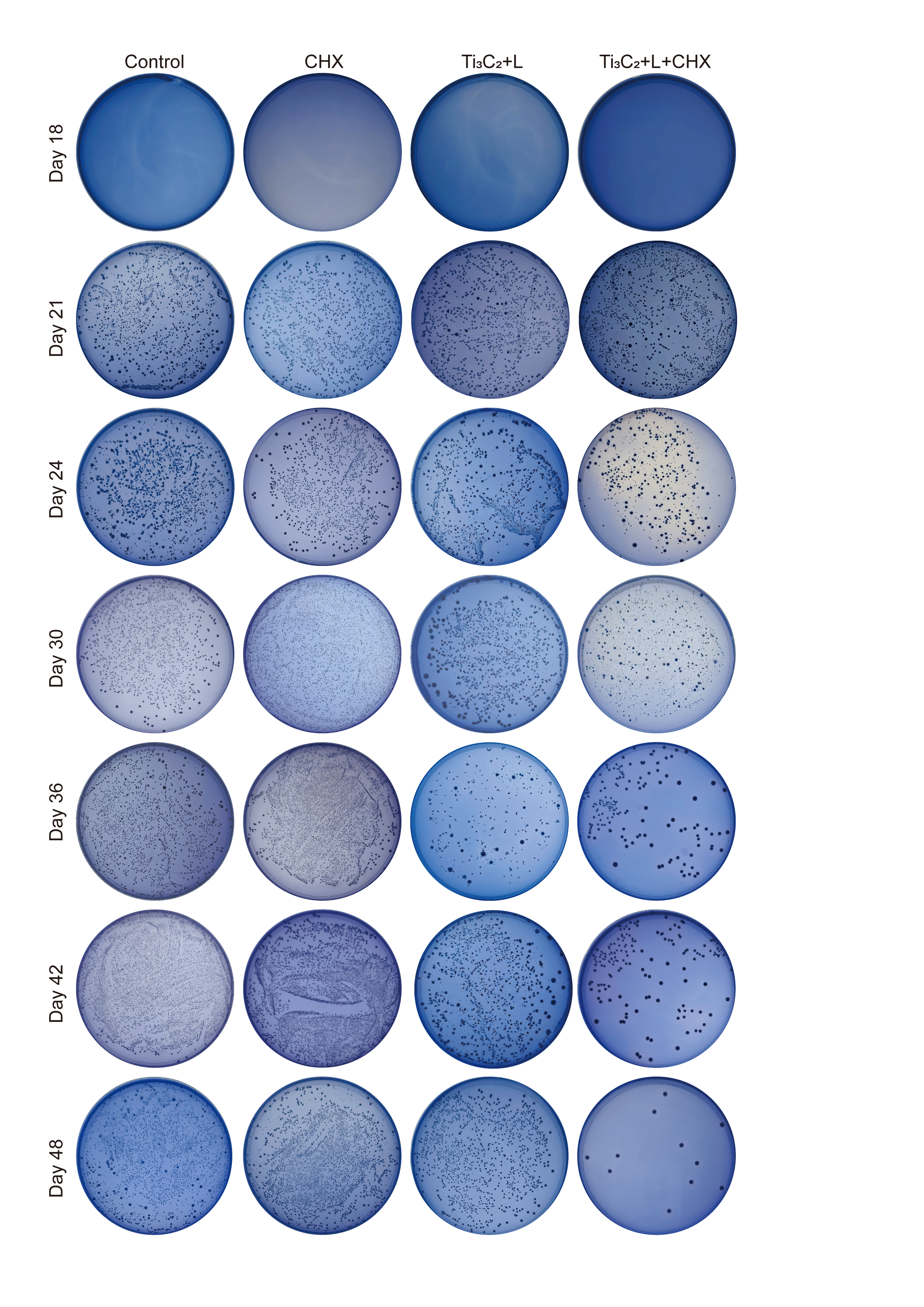  **Figure S17.** Representative images of surviving bacterial colonies on MSB agar plates with different treatments at 18, 21, 24, 30, 36, 42, and 48 days.  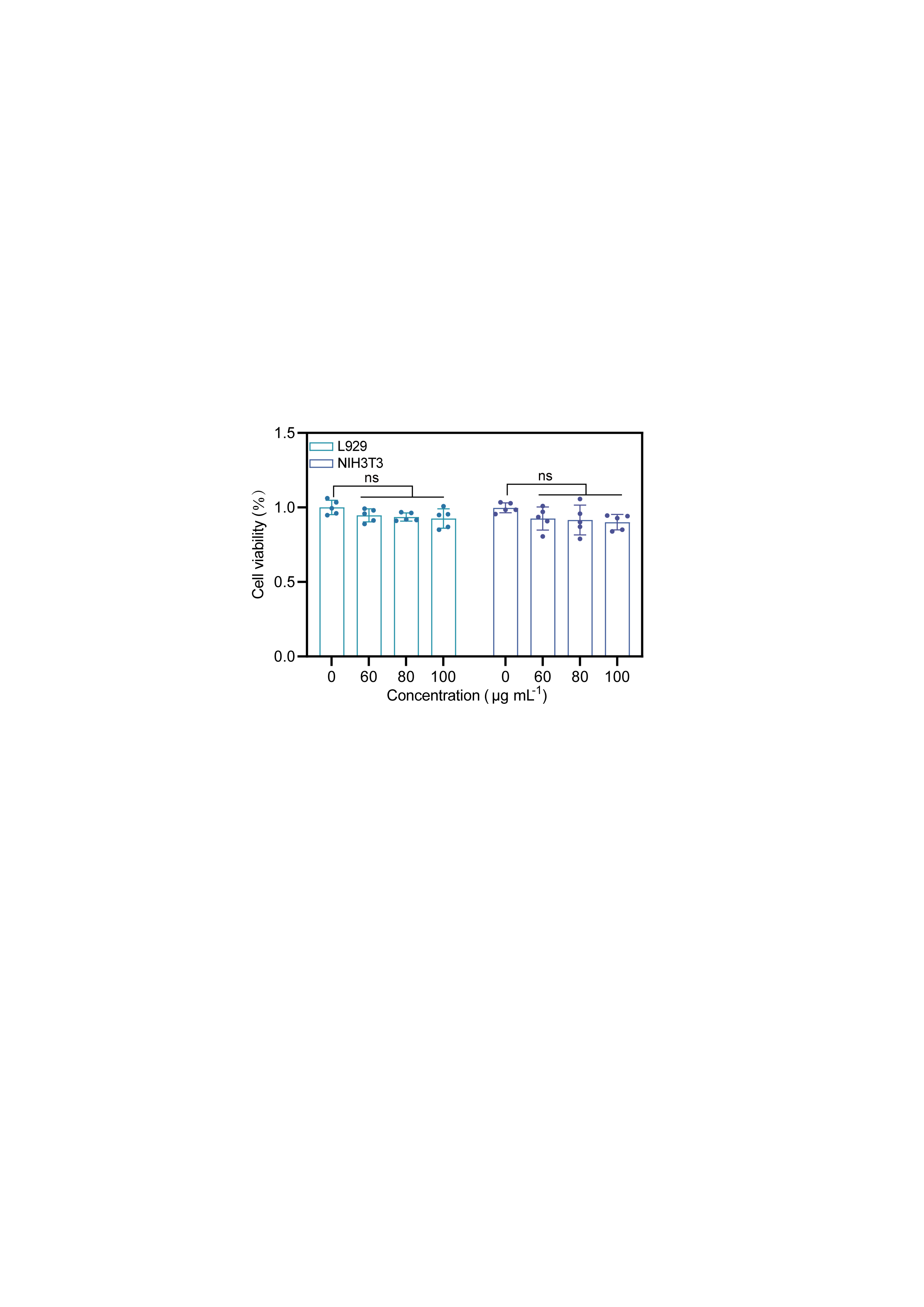  **Figure S18.** Cell viability of normal cells (L929 and NIH3T3) upon treatment with Ti_3_C_2_ at different concentrations (0–100 μg mL^-1^ Ti_3_C_2_; ns, *p* > 0.05).  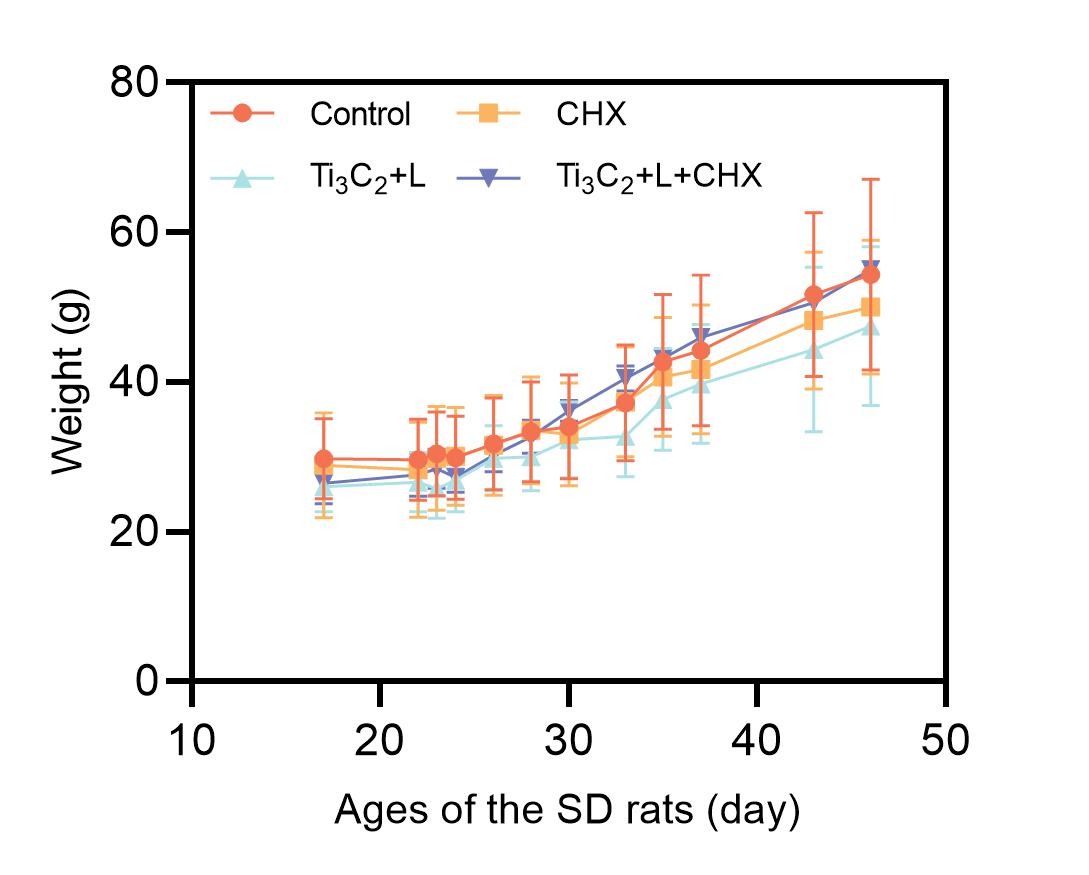  **Figure S19.** Body weight changes of rats with different treatments.  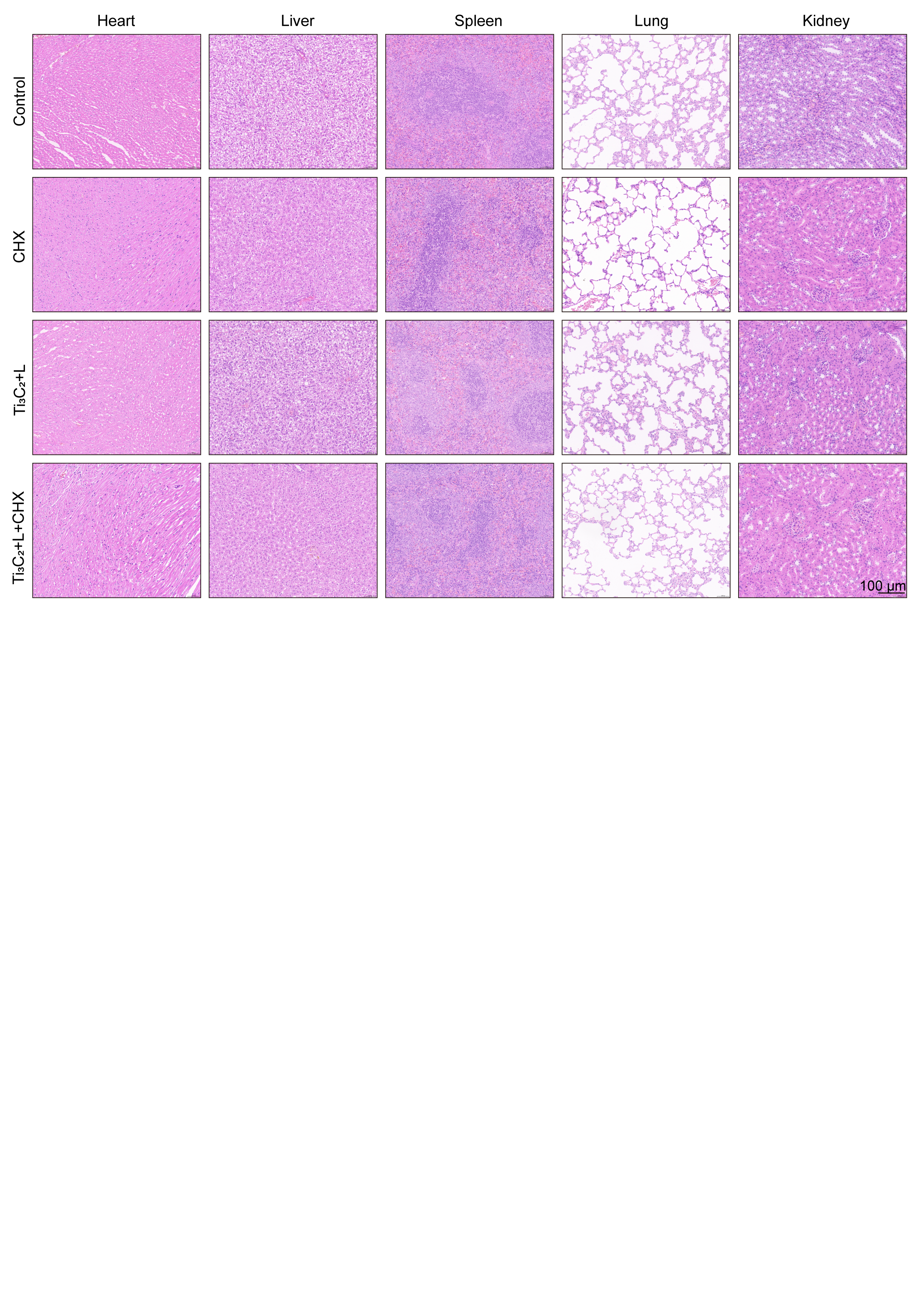  **Figure S20.** Histology of the main organs (heart, liver, spleen, lung, and kidney) through H&E staining with the treatments noted (scale bar, 100 μm). |  |
| --- | --- |

**Table S1 Differential expression level of proteins and genes with their primers.**

| Gene | Forward Primer (5’-3’) | Reversed Primer (5’-3’) |
| --- | --- | --- |
| *gtfB* | AGCAATGCAGCCARTCTACAAAT | ACGAACTTTGCCGTTATTGTC |
| *gtfD* | CACAGGCAAAAGCTGAATTAACA | AATGGCCGCTAAGTCAACAG |
| *dnak*  *dnaJ*  *hrcA* | TGACCGCAACACTACTATTCCA  ATCAATATGGATACCCAAACGC  CGGCGATTACGCTTGATGT | GTCTTGTTATCCGCTGCCATT  CAGCAGGAATCTTGACAGAAACT  CTTGGGAATGGCGAACTGG |
| *grpE* | AGAAGTAGAAGCAACTGAGCCTACA | TTCAGCATGAGCACGCAAATA |
| 16s *rRNA* | GGGAATCTTCGGCAATGGA | GGCTGCTGGCACGTAGTTAG |

**Table S2.** Oligonucleotide probes used for FISH

| Fluorescein | Fluorescence | Excitation/Emission | Nucleotide Sequence (5’-3’) | Target Gene |
| --- | --- | --- | --- | --- |
| TRITC/CY3 (CY3) | Red | 561nm/590 nm | TGCTGCAGCTGTTGGTTCGTTGAC | HSP |

**Table S3.** The composition of each simulation system.

|  | DPPC | K+ | Cl- | H_2_O | Ti_3_C_2_ (10*10*1 unit) |
| --- | --- | --- | --- | --- | --- |
| System | 204 | 21 | 21 | 10000 | 1 |
